# Acetylcholine Neurotransmitter Receptor Densities in the Striatum of Hemiparkinsonian Rats Following Botulinum Neurotoxin-A Injection

**DOI:** 10.3389/fnana.2018.00065

**Published:** 2018-08-10

**Authors:** Teresa Mann, Karl Zilles, Felix Klawitter, Markus Cremer, Alexander Hawlitschka, Nicola Palomero-Gallagher, Oliver Schmitt, Andreas Wree

**Affiliations:** ^1^Rostock University Medical Center, Institute of Anatomy, Rostock, Germany; ^2^Research Centre Jülich, Institute of Neuroscience and Medicine INM-1, Jülich, Germany; ^3^Department of Psychiatry, Psychotherapy and Psychosomatics, Medical Faculty, RWTH Aachen, Aachen, Germany; ^4^JARA-Translational Brain Medicine, Aachen, Germany

**Keywords:** receptors, acetylcholine, hemiparkinsonian rat model, Botulinum neurotoxin-A, basal ganglia, Parkinson's disease

## Abstract

Cholinergic neurotransmission has a pivotal function in the caudate-putamen, and is highly associated with the pathophysiology of Parkinson's disease. Here, we investigated long-term changes in the densities of the muscarinic receptor subtypes M_1_, M_2_, M_3_ (mAchRs) and the nicotinic receptor subtype α_4_β_2_ (nAchRs) in the striatum of the 6-OHDA-induced hemiparkinsonian (hemi-PD) rat model using quantitative *in vitro* receptor autoradiography. Hemi-PD rats exhibited an ipsilateral decrease in striatal mAchR densities between 6 and 16%. Moreover, a massive and constant decrease in striatal nAchR density by 57% was found. A second goal of the study was to disclose receptor-related mechanisms for the positive motor effect of intrastriatally injected Botulinum neurotoxin-A (BoNT-A) in hemi-PD rats in the apomorphine rotation test. Therefore, the effect of intrastriatally injected BoNT-A in control and hemi-PD rats on mAchR and nAchR densities was analyzed and compared to control animals or vehicle-injected hemi-PD rats. BoNT-A administration slightly reduced interhemispheric differences of mAchR and nAchR densities in hemi-PD rats. Importantly, the BoNT-A effect on striatal nAchRs significantly correlated with behavioral testing after apomorphine application. This study gives novel insights of 6-OHDA-induced effects on striatal mAchR and nAchR densities, and partly explains the therapeutic effect of BoNT-A in hemi-PD rats on a cellular level.

## Introduction

Acetylcholine (Ach) effects are mediated via metabotropic G-protein coupled muscarinic receptors (mAchRs) and ionotropic nicotinic receptors (nAchRs). They are important for cognitive functions such as working, episodic and spatial memory, or attention (Hefco et al., 2004; Newman et al., 2012). In the striatum (caudate-putamen, CPu), complex bidirectional interactions of the cholinergic and dopaminergic systems are fundamental for normal functioning (Calabresi et al., 2000; Cragg, 2006; Goldberg et al., 2012). Both nAchRs (Giorguieff et al., 1976; Rapier et al., 1988; Grady et al., 1992; El-Bizri and Clarke, 1994; Sharples et al., 2000; Wonnacott et al., 2000; Zhou et al., 2001) and mAchRs (Westfall, 1974; Zhang et al., 2002; Zhang and Sulzer, 2004; Threlfell et al., 2010) modulate dopamine (DA) release from synaptic terminals.

The nAChRs (such as the α_4_β_2_ subtype) as well as mAChRs (M_1_-M_5_ subtypes) interact in a complex manner to modulate synaptic plasticity in the striatum. M_1_ receptors presynaptically located on medium spiny neurons (MSNs) inhibit GABA release (Sugita et al., 1991). To a lesser degree, glutamate release from glutamatergic terminals is also inhibited by activation of other muscarinic presynaptic receptors. Since the effect on GABA release dominates, the netto effect of M_1_ receptor activation is increased excitability (Sugita et al., 1991). M_2_ and M_3_ receptors both regulate glutamatergic release from cortical nerve terminals (Calabresi et al., 1998; Pakhotin and Bracci, 2007). Activation of M_4_ receptors, which often form heterodimers with the excitatory D_1_ receptor on MSNs (Ince et al., 1997) results in the inhibition of these neurons. Presynaptic M_2_ and M_4_ receptors modulate glutamate release from corticostriatal afferents and GABA release from GABAergic interneurons (Benarroch, 2012). Moreover, M_2_ and M_4_ receptors were both found as autoreceptors on cholinergic interneurons in the striatum (Zhou et al., 2003). Also nAChRs are expressed on cholinergic interneurons as well as on corticostriatal and nigrostriatal terminals. They modulate glutamate release (Zhou et al., 2003). However, the precise functions of nAChRs and mAChRs subtypes are still subject of ongoing research.

In Parkinson's disease (PD) the primary neuropathological characteristic is the dopaminergic denervation of the CPu caused by progressive dopaminergic cell death in the substantia nigra pars compacta (SNpc) (Hornykiewicz, 1963; Bernheimer et al., 1973). The resulting DA deficit triggers its cardinal clinical symptoms including tremor, rigidity, bradykinesia and postural instability (Cutson et al., 1995). Further, the absent inhibition of tonically active cholinergic interneurons by DA results in relative hypercholinism (Spehlmann and Stahl, 1976; Aosaki et al., 2010), which additionally worsens the pathological motor manifestations (Marti et al., 1999; Pisani et al., 2003; Ding et al., 2006). Consequently, disturbances in cholinergic neurotransmission are associated with symptoms of PD (Lester et al., 2010).

Symptomatic pharmacological treatments of PD are DA replacement therapy with L-Dopa (Carlsson et al., 1957; Duvoisin, 1967; Volpato et al., 2016), administration of DA receptor agonists, of catecholamine-O-methyl transferase inhibitors, and of anticholinergics drugs including mAchR antagonists (Horstink et al., 2006a,b; Langmead et al., 2008). Anticholinergic substances have anti-parkinsonian effects on motor dysfunction (Brooks, 1998; Lees, 2005). The systemic administration of anticholinergica is, however, accompanied by massive side effects (Katzenschlager and Lees, 2002; Fernandez, 2012). We recently demonstrated that the local injection of the anticholinergic Botulinum neurotoxin-A (BoNT-A) into the CPu is a potent new experimental therapy in 6-hydroxdopamine (6-OHDA)-induced hemiparkinsonian (hemi-PD) rats. Peripheral and central side effects of systemically applied anticholinergica are avoided in this approach (Wree et al., 2011; Antipova et al., 2013; Wedekind et al., 2018).

Experimental animal models that mimic aspects of PD by destruction of the nigrostriatal pathway are frequently used in PD's research and therapy testing. One is the hemi-PD rat model, which is generated by unilateral stereotaxic injection of the neurotoxin 6-OHDA into the medial forebrain bundle (MFB) (Ungerstedt, 1968; Ungerstedt and Arbuthnott, 1970; Schwarting and Huston, 1996; Blandini et al., 2000; Duty and Jenner, 2011; Tieu, 2011). The resulting dopaminergic depletion in hemi-PD rats affects motor behavior as seen in the apomorphine-induced rotation test (Ungerstedt et al., 1969). We previously demonstrated that apomorphine-induced contralateral rotations of hemi-PD rats are abolished by ipsilateral injection of BoNT-A up to 6 months (Wree et al., 2011; Antipova et al., 2013, 2017; Mann et al., 2018a,b). Moreover, BoNT-A does not have cytotoxic effects in the rat brain (Mehlan et al., 2016), and does not impair cognition but reduces anxiety in naïve rats (Holzmann et al., 2012). BoNT-A inhibits Ach release from presynaptic terminals by cleavage of the synaptosome-associated glycoprotein of 25-kDa (SNAP25), and thus prevents cholinergic hyperactivity (Coffield et al., 1994; Caleo et al., 2009). However, the molecular and cellular mechanisms of the observed behavioral effects of BoNT-A on hemi-PD rats have not yet been fully examined.

To explore the role of cholinergic receptors and its changes after DA deprivation and following BoNT-A injection, we comprehensively examined striatal receptor densities of mAchRs (M_1_, M_2_, M_3_, M_4_ subtypes) and nAchRs (α_4_β_2_ subtype) up to a survival time of 9 months using quantitative *in vitro* receptor autoradiography. First, we analyzed time-dependent changes in receptor densities in 6-OHDA-induced hemi-PD rats relative to controls. Then the long-term effects of intrastriatal BoNT-A injections in naïve rats were investigated. To our knowledge, this is the first study analyzing long-term effects of 6-OHDA and intrastriatal BoNT-A injections.

## Materials and methods

### Animals

One hundred and twenty-eight male Wistar rats (strain Crl:WI BR) aged 3 months and weighing 250–280 g were obtained from Charles River WIGA (Sulzfeld, Germany). Animals were housed in a temperature-controlled room (22 ± 2°C) under a fixed 12 h light/12 h dark cycle and had free access to food and water 24 h a day. Animal treatment was in line with legal obligations of the animal welfare act and all animal experiments were approved by the state Animal Research Committee of Mecklenburg-Western Pomerania (LALLF M-V/TSD/7221.3-1.1-003/13).

### Stereotactic interventions

Anesthesia was performed via intraperitoneal (i.p.) injection of a mixture of ketamine (50 mg/kg body weight) and xylazine (4 mg/kg body weight). Rats were operated under aseptic conditions at a weight of 285–305 g. Animals were fixed in a rat stereotactic apparatus (Kopf, Tujunga, CA, USA) and 6-OHDA solution was injected evenly over 4 min via a 26 gauge 5 μl Hamilton syringe into the right MFB. The coordinates of the 6-OHDA injection with reference to bregma were: anterior-posterior = −2.3 mm, lateral = −1.5 mm and ventral = −9.0 mm (Paxinos and Watson, 2007). Hemi-PD was induced by the unilateral injection of 4 μl of 6-OHDA (24 μg) (Sigma-Aldrich, St. Louis, MO) dissolved in 0.1 M citrate buffer. Application of BoNT-A (lot No. 13028A1A; List, Campbell, CA; purchased via Quadratech, Surrey, UK) or of vehicle was carried out 6 weeks after 6-OHDA lesion. BoNT-A was handled and stored according to the precautions given by the manufacturer. The coordinates of the two BoNT-A or vehicle injections with reference to bregma were: anterior-posterior = +1.3 mm/−0.4 mm, lateral = −2.6 mm/−3.6 mm and ventral = −5.5 mm/−5.5 mm (right CPu) (Paxinos and Watson, 2007). For details of the different control and experimental groups see Table 1.

**Table 1:** Details of the different control and experimental groups and their survival times.

**Groups**	**Characteristics**	**Survival times**
1. Controls **(C)**	Unoperated naive rats	300 g body weight + 6 weeks **(C)** (*n* = 7)
2. 6-OHDA only **(L)**	Single injection of 24 μg 6-OHDA into the right medial forebrain bundle	Weeks after 6-OHDA **(L3W)** (*n* = 7) 6 weeks after 6-OHDA **(L6W)** (*n* = 7) 6 weeks + 1 month after 6-OHDA **(L6W1M)** (*n* = 7) 6 weeks + 3 months after 6-OHDA **(L6W3M)** (*n* = 6) 6 weeks + 6 months after 6-OHDA **(L6W6M)** (*n* = 6) 6 weeks + 9 months after 6-OHDA **(L6W9M)** (*n* = 6)
3. BoNT-A only **(B)**	Two injections of 1 ng BoNT-A [solved in phosphate-buffered saline (PBS) supplemented with 0.1% bovine serum albumin (BSA)], each at different sites within the right CPu	Weeks after BoNT-A **(B2W)** (*n* = 7) 1 month after BoNT-A **(B1M)** (*n* = 6) months after BoNT-A **(B3M)** (*n* = 6) 6 months after BoNT-A **(B6M)** (*n* = 7) 9 months after BoNT-A **(B9M)** (*n* = 7)
4. 6-OHDA + BoNT-A **(LB)**	Single injection of 24 μg 6-OHDA into the right medial forebrain bundle followed by 2 × 1 ng BoNT-A	6 weeks after 6-OHDA + 1 month after BoNT-A **(LB6W1M)** (*n* = 9) 6 weeks after 6-OHDA + 3 months after BoNT-A **(LB6W3M)** (*n* = 8) 6 weeks after 6-OHDA + 6 months after BoNT-A **(LB6W6M)** (*n* = 8) 6 weeks after 6-OHDA + 9 months after BoNT-A **(LB6W9M)** (*n* = 8)
5. 6-OHDA + vehicle **(LV)**	Single injection of 24 μg 6-OHDA into the right medial forebrain bundle + 2 × 1 μl vehicle (PBS + 0.1% BSA)	6 weeks after 6-OHDA + 1 month after vehicle **(LV6W1M)** (*n* = 6) 6 weeks after 6-OHDA + 3 months after vehicle **(LV6W1M)** (*n* = 8)

### Apomorphine rotation test

Successful 6-OHDA lesion as well as the effect of BoNT-A on motoric behavior were verified by the drug-induced apomorphine rotation test (Ungerstedt and Arbuthnott, 1970). All animals were examined in the apomorphine rotation test 1 month post 6-OHDA lesioning and repetitively over a time period of up to 9 months. Animals were placed in a self-constructed rotometer device modified according to Ungerstedt and Arbuthnott (1970) 5 min after apomorphine injection (0.25 mg/kg, i. p.). Full rotations of 360° were counted over 40 min and the mean rotation per minute was calculated (anti-clockwise: +, clockwise: –).

### Tissue processing

For tissue processing brains were dissected, frozen in isopentane (−80°C) and cut in frontal sections (20 μm) on a cryostat (Leica Mikrosystems, Wetzlar, Germany). Mounting was performed on gelatin-coated and pre-cooled (−20°C) glass slides followed by a drying procedure (20–30 min, +35°C). Sections were obtained from 7 predefined levels, which were selected to cover the entire rostro-caudal extent of the CPu. The coordinates with reference to bregma were: level 1: +1.56 mm, level 2: −0.36 mm, level 3: −0.84 mm, level 4: −2.16 mm, level 5: −2.52 mm, level 6: −3.60 mm, and level 7: −5.20 mm (Paxinos and Watson, 2007). Each level consisted of immediately adjacent sections used for the visualization of the different receptor types, or of cell bodies.

### Receptor autoradiography

Autoradiography was performed according to published protocols (Zilles et al., 1991a,b,c, 2002a,b). The M_1_ receptor was labeled with [^3^H]pirenzepine (M_1_ antagonist), the agonistic binding site of the M_2_ receptor with [^3^H]oxotremorine-M, and its antagonistic binding site with [^3^H]AF-DX 384. The antagonistic ligand [^3^H]AF-DX 384 binds not only to M_2_ receptors, but also to M_4_ receptors in a regional specific manner (Valuskova et al., 2018). In the mouse striatum, almost 80% of this binding is to M_4_ receptors. The M_3_ receptor was labeled with [^3^H]4-DAMP (M_3_ antagonist) and the α_4_β_2_ nicotinic receptor with [^3^H]epibatidine (α_4_β_2_ agonist). All ligands were purchased from Perkin Elmer (Rodgau, Germany). Autoradiographic processing of the sections was performed in 3 main steps: rehydration and elimination of endogenous ligands, incubation with the respective tritiated ligand in the absence (total binding) or presence (nonspecific binding) of a specific non-radioactive ligand as displacer, and a final washing step to remove non-bound ligand and buffer salts. Sections were then dried and co-exposed with different plastic standards (representing different radioactivity concentrations) against ß-sensitive films (Kodak, PerkinElmer LAS GmbH, Germany) for 9 weeks ([^3^H]AF-DX 384, [^3^H]4-DAMP), 12 weeks ([^3^H]pirenzepine) or 15 weeks ([^3^H]epibatidine, [^3^H]oxotremorine-M). Resulting autoradiographs were developed using a Hyperprocessor (Amersham Biosciences, Amersham, UK; now: GE Healthcare Europe GmbH, Freiburg, Germany) and digitized with a CCD-camera (Zeiss, Carl Zeiss MikroImaging GmbH, Göttingen, Germany). Details of autoradiographic processing for each receptor are shown in Table 2.

**Table 2:** List of analyzed receptors with the respective specific ligands, nonradioactive displacers, washing, and incubation details.

**Receptor**	**Ligand**	**Displacer**	**Incubation buffer**	**Pre-incubation**	**Main incubation**	**Final rinsing**
M_1_	[^3^H]-Pirenzepine 1.0 nM	Pirenzepine μm	Modified Krebs buffer (pH 7.4), 5.6 mM KCl, 30.6 mM NaCl, 1.2 mM MgSO_4_, 1.4 mM KH_2_PO_4_, 5.6 mM D-Glucose, 5.2 mM NaHCO_3_, 2.5 mM CaCl_2_	15 min, 4°C	60 min, 4°C	1) × 1 min, 4°C 2) 1 s in distilled water, 22°C
M_2_ (agonist)	[^3^H]-Oxotremorine-M 1.7 nM	Carbachol 10μm	20 mM HEPES-Tris (pH 7.5), 10 nM MgCl_2_, 300 nM Pirenzepine	20 min, 22°C	60 min, 22°C	1) × 2 min, 4°C 2) 1 s in distilled water, 22°C
M_2_ (antagonist)	[^3^H]-AF-DX 384 5 nM	Atropinesulfate 100μm	Modified Krebs buffer (pH 7.4) 4.7 mM KCl 120 mM NaCl, 1.2 mM MgSO_4_, 1.2 mM KH_2_PO_4_, 5.6 mM D-Glucose, 25 mM NaHCO_3_, 2.5 mM CaCl_2_	15 min, 22°C	60 min, 22°C	1) × 4 min, 4°C 2) 1 s in distilled water, 22°C
M_3_	[^3^H]-DAMP 1 nM	Atropinesulfate 10μm	50 mM Tris-HCl (pH 7.4), 0.1 mM PMSF, 1 mM EDTA	15 min, 22°C	45 min, 22°C	1) × 5 min, 4°C 2) 1 s in distilled water, 22°C
α_4_β_2_	[^3^H]-Epibatidine 0.5 nM	Nicotine 100μM	15 mM Hepes (pH 7.5), 120 mM NaCl, 5.4 mM KCl, 0.8 mM MgCl_2_, 1.8 mM CaCl_2_	20 min, 22°C	90 min, 22°C	1) 1 × 5 min, 4°C 2) 1 s in distilled water, 22°C

### Histology

Alternating sections were processed for the visualization of cell bodies. These sections were postfixed in 3.7% paraformaldehyde (20–45 min). Staining of cell bodies was performed with 0.1% cresyl-violet solution (7–12 min, 60°C; cresyl-violet acetate SIGMA C1791-5G). These sections were used as histological reference during the delineation of the CPu in the autoradiographs.

### Image processing

The software MCID Analysis v7.0 (InterFocus imaging Ltd, Linton, UK) (http://www.mcid.co.uk/) was used for measurement of receptor densities. Digitized autoradiographs were loaded and regions of interest (ROIs) were defined manually by comparison with the neighbouting cell-body stained sections. For each ROI, the average and background-corrected gray values were extracted. For densitometric analysis the gray values of all processed sections were transformed into receptor densities (in fmol/mg protein) by means of standard curves derived from the co-exposed plastic standards. In the ensuing linearized autoradiographs, which can be color coded for visualization purposes, the receptor density in fmol/mg protein of each pixel can be calculated according to:

(1) $$\begin{array}{r} C_{b}=\;\frac{R}{E\cdot B\cdot W_{b}\cdot S_{a}}\;\cdot \;\frac{K_{D}+L}{L} \end{array}$$

where *R* is the radioactivity concentration in counts per minute [cpm], *C*_*b*_ is B_max_ and thus the binding site concentration in fmol/mg, *E* is the efficiency of the scintillation counter (depends on the actual counter), *B* is a constant representing the number of decays per unit of time and radioactivity [Ci/min], *W*_*b*_ the known equivalent protein weight of a standard [mg], *S*_*a*_ the specific activity of the ligand [Ci/mmol], *K*_*D*_ the dissociation constant of the ligand [nM], and *L* the free concentration of the ligand during incubation [nM]. Displayed receptor density values are the area-weighted means of all ROIs of an animal containing the CPu. Since the tritiated ligands do not only bind to a single receptor type, the *K*_*D*_ value for each ligand is important to reach the necessary selectivity for demonstration of a single receptor type. Therefore, the ligand concentration in the incubation buffer was set at 50% of the *K*_*D*_ value for the dissociation of the ligand-receptor binding. This results in a high specificity of receptor labeling.

### Statistical analysis

For all statistical observations, IBM SPSS Statistics version 20.0 was used. Gaussian distribution of receptor density values was tested with the Kolmogorov-Smirnov test. Then, data was analyzed with a between-subject Univariate General Linear Model (*post-hoc* ANOVA analysis of variance). The dependent variable was “receptor density” and the covariate was the respective treatment group separately for the left and right hemispheres not considering survival time followed by Bonferroni correction with the factor group [*df* = 9; (M_1_ receptor: *F* = 1.199; M_2_ (agonist binding) receptor: *F* = 1.847, M_2_ (antagonist binding) receptor: *F* = 19.180, M_3_ receptor: *F* = 22.787, nAch receptor: *F* = 148.214)]. Correlations between relative receptor density and apomorphine-induced rotations as well as time-dependent correlations of right-left differences were analyzed with linear regression followed by a two-sided Pearson correlation test. Possible significant differences in body weight between 2 groups were analyzed using factorial ANOVA. *p* < 0.05 was considered statistically significant.

## Results

Quantitative *in vitro* receptor autoradiography was performed to analyze the mean densities of mAchRs (M_1_, M_2_, M_3_) and nAchRs (α_4_β_2_) longitudinally between 3 weeks and 9 months post lesion in 5 experimental groups (for details see Table 1). Mean receptor densities and interhemispheric differences) of mAchRs and nAchRs are presented in Table 3.

**Table 3:** Summary of the mean receptor densities in the CPu for all analyzed receptors in fmol/mg protein ± SD and the interhemispheric difference (mean ± SD) (in %) for the 5 groups averaged over all post-lesion survival times [controls (C), hemi-PD rats (L), BoNT-A only rats (B), hemi-PD + BoNT-A rats (LB) and hemi-PD + vehicle rats (LV)].

**Receptor**	**Mean C**	**Mean L**	**Mean B**	**Mean LB**	**Mean LV**
**M**_1_					
Left hemisphere Right hemisphere Change (relative to left)	5,149 ± 2,335 5,263 ± 2,328 98 ± 1%	5,263 ± 1,190 4,772 ± 2,328 91 ± 5%	5,130 ± 1,414 5,083 ± 1,443 99 ± 6%	5,073 ± 1,328 4,905 ± 1,288 97 ± 6%	4,268 ± 1,591 3,958 ± 1,417 93 ± 5%
**M**_2_ **(AGONIST BINDING)**					
Left hemisphere Right hemisphere Change (relative to left)	924 ± 82 974 ± 109 105 ± 7%	938 ± 70 887 ± 77 96 ± 7%	965 ± 88 984 ± 82 98 ± 11%	978 ± 85 936 ± 84 96 ± 8%	862 ± 72 828 ± 81 96 ± 6%
**M**_2_ **AND M**_4_ **(ANTAGONIST BINDING)**					
Left hemisphere Right hemisphere Change (relative to left)	3894 ± 363 3,859 ± 352 99 ± 2%	3,405 ± 190 2,877 ± 218^***^ 84 ± 5% ^###^	3,337 ± 197^*^ 3,085 ± 254^***^ 92 ± 9%	2,997 ± 216^***^ 2,595 ± 271^**^ 87 ± 9% ^###^	3,197 ± 180^*^ 2,737 ± 264^***^ 86 ± 6%
**M**_3_					
Left hemisphere Right hemisphere Change (relative to left)	7,274 ± 709 7,368 ± 757 101 ± 3%	4,999 ± 419^***^ 4,717 ± 417^***^ 94 ± 4%	5,398 ± 541^***^ 5,473 ± 486^***^ 101 ± 7%	4,853 ± 572^***^ 4,725 ± 613^***^ 97 ± 7%	5,316 ± 773^***^ 5,239 ± 745^***^ 99 ± 5%
α_4_β_2_					
Left hemisphere Right hemisphere Change (relative to left)	392 ± 35 382 ± 23 98 ± 7%	316 ± 25^***^ 136 ± 20^***^ 43 ± 5% ^###^	335 ± 23^*^ 354 ± 22 106 ± 7%	331 ± 31^*^ 175 ± 23^***^ 53 ± 7%	309 ± 20^***^ 135 ± 18^***^ 44 ± 8%

Asterisks indicate significance of a hemisphere to the respective side of the controls

* *p < 0.05*,

** *p < 0.01*,

*** p < 0.001. Rhombs display interhemispheric significance within one group

### *p < 0.001. Significance was calculated with a between-subject Univariate General Linear Model (post-hoc ANOVA analysis of variance) using the mean receptor density of every treatment group separately for the left and right hemisphere*.

### 6-OHDA lesion effect on rotational behavior and body weight

The apomorphine-induced rotation test was performed 3 weeks post 6-OHDA lesion and 1 month post BoNT-A injection to functionally confirm dopaminergic deafferentiation after 6-OHDA lesion and the effect of BoNT-A on motoric behavior. All hemi-PD rats (groups L, LB, LV) demonstrated a contralateral net rotation of about 8 min^−1^ before the BoNT-A or vehicle injections. Intrastriatal BoNT-A injection in hemi-PD rats (group LB) significantly abolished contralateral rotations for up to 3 months. Vehicle injection in hemi-PD rats (group LV) did not affect the rotational behavior, as previously shown (Mann et al., 2018b). TH-immunoreactive staining in the SNpc of both hemispheres revealed no side differences in control rats, a distinct loss of TH-positive neurons ipsilateral to the 6-OHDA lesion, and no additive effect of BoNT-A as previously shown (Mann et al., 2018b). Body weight was not significantly different after BoNT-A administration (analyzed in groups LB and LV) 1 month and 3 months post intervention. Results expressed as weight ± SD were LB6W1M: 414 ± 44 g, LV6W1M: 457 ± 35 g, *p* = 0.069; LB6W3M: 476 ± 42 g, LV6W3M: 495 ± 52 g, *p* = 0.460.

### Muscarinic M_1_ receptors

**Figure 1:**
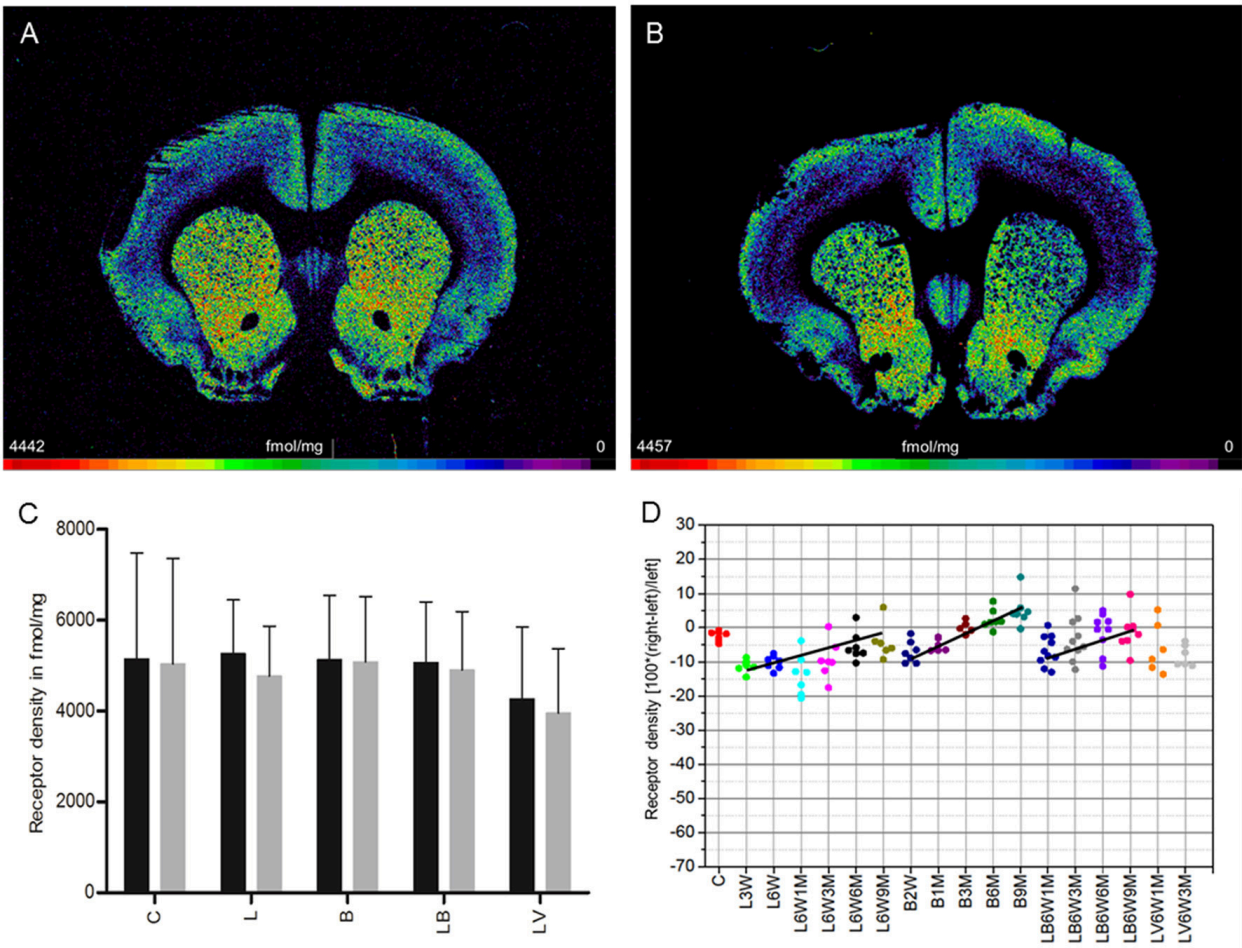
**(A,B)** Contrast-enhanced color-coded images showing the regional distribution of M_1_ receptor density labeled with [^3^H]pirenzepine in a control rat **(A,B)** after 6-OHDA lesion (group L6W1M) in a control rat **(A)** and after 6-OHDA lesion (group L6W1M) **(B)**. **(C)** Mean receptor density (fmol/mg protein; averaged over all post-lesion survival times) in the left/contralateral (black column) and right/ipsilateral hemispheres (gray column) of the 5 groups: C, control; L, 6-OHDA lesion; B, BoNT-A only; LB, L + BoNT-A; LV. L + vehicle. All data is expressed as means ± SD. **(D)** Scatter plots and regression analyses of the right-left differences of M_1_ receptor density for all 18 groups and time points (five groups with different survival times); see Table 1 for explanation of abbreviations. Significant regressions are labeled by a continuous line.

In the CPu of control rats (group C), M_1_ receptors showed a mean concentration of 5,092 ± 2,331 fmol/mg (mean ± SD) without significant interhemispheric differences (Figures 1A,C). 6-OHDA lesion (group L) non-significantly decreased interhemispheric M_1_ receptor density by about 9% ipsilateral to the lesion, the contralateral side remained at a level comparable to that of controls (Figures 1B,C). Notably, the potential effect of the 6-OHDA lesion on ipsilateral M_1_ receptors was more obvious in the first 3 months after 6-OHDA lesion (L3W −11.2%, L6W −10.2%, L6W1M −13.8%, L6W3M −9.5%, L6W6M −5.4%, L6W9M −4.1%) (*R*^2^ = 0.24083, *p* = 0.001) (Figure 1D).

Injection of only BoNT-A (group B) did not result in interhemispheric differences when densities were averaged over all analyzed survival times (Figure 1C). Considering the post injection time course after application of BoNT-A in this group, there was an ipsilateral reduction of M_1_ receptor density of −7.0% 2 weeks after BoNT-A. This reduction significantly decreased (*R*^2^ = 0.71129, p = 0.001) with longer post injection survival (1 month −5.1%, 3 months −0.3%), and changed into an increase (6 months +2.4%, 9 months +5.1%) (Figure 1D).

BoNT-A injection in hemi-PD rats (group LB) did not significantly affect the right-left difference of M_1_ receptors when densities were averaged over all survival times (Figure 1C). Notably, the interhemispheric difference of group LB was reduced for all analyzed time points (LB6W1M −7.0%, LB6W3M −3.3%, LB6W6M −2.0% and LB6W9M −1.4%) (*R*^2^ = 0.15267, *p* = 0.022) (Figure 1D). Vehicle injection in hemi-PD rats (group LV) did not affect the 6-OHDA-induced imbalance of M_1_ receptor density (Figures 1C,D) as compared to group L.

To analyze a possible relation between interhemispheric differences and apomorphine-induced rotations and the effect of BoNT-A and Sham injection in this context, we correlated relative M_1_ receptor density with apomorphine-induced rotations for groups LB and LV and did not find a significant correlation (*R*^2^ = 0.009983, *p* = 0.7231).

### Muscarinic M_2_ receptor (agonist binding)

**Figure 2:**
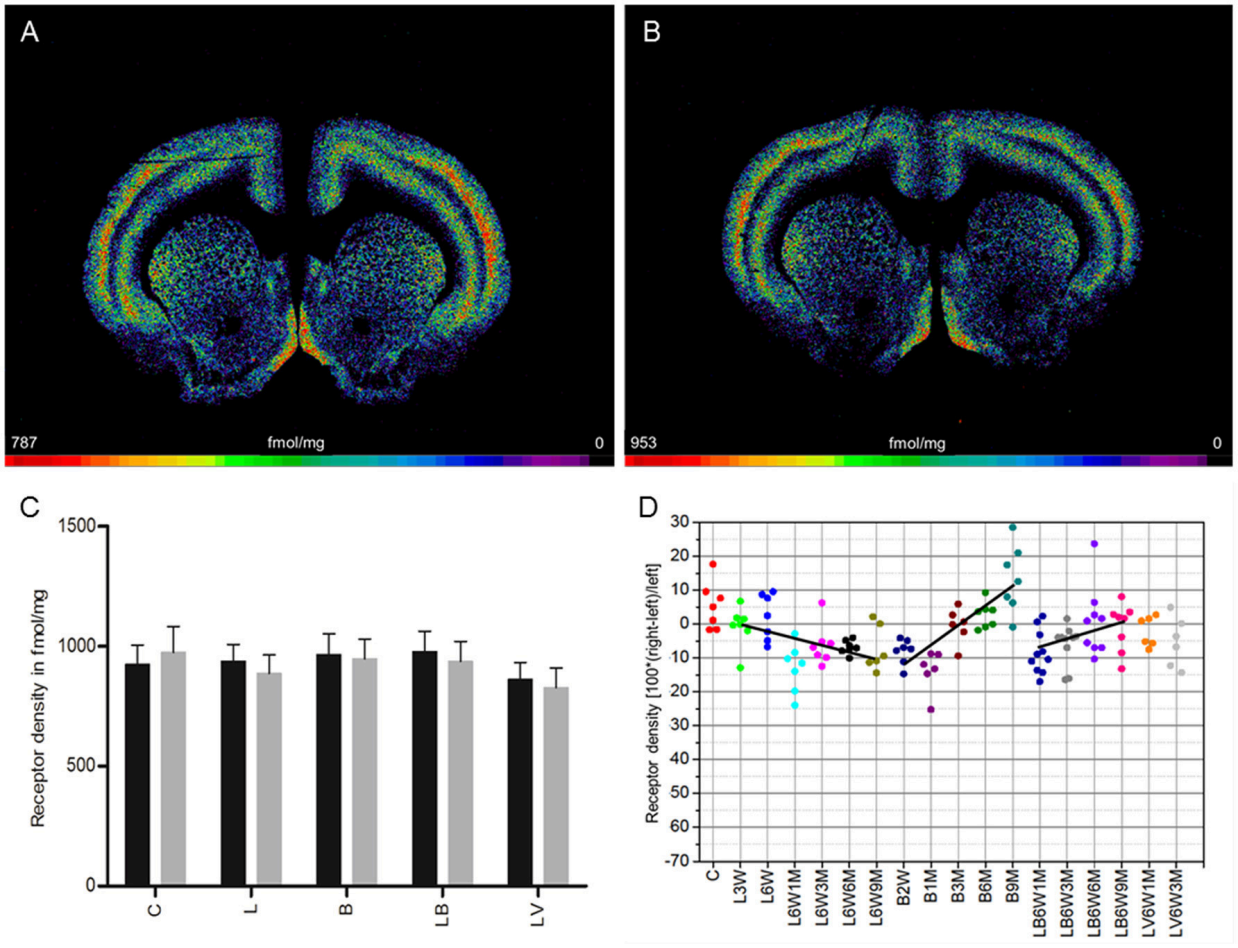
**(A,B)** Contrast-enhanced color-coded images showing the regional distribution of M_2_ receptor density labeled with the agonist [^3^H]oxotremorine-M in a control rat **(A,B)** after 6-OHDA lesion (group L6W1M) in a control rat **(A)** and after 6-OHDA lesion (group L6W1M) **(B)**. **(C)** Mean receptor density (fmol/mg protein; averaged over all post-lesion survival times) in the left/contralateral (black column) and right/ipsilateral hemispheres (gray column) of the 5 groups (see Figure 1). All data is expressed as means ± SD. **(D)** Scatter plots and regression analyses of the right-left differences of M_2_ receptor density for all 18 groups and time points (five groups with different survival times); see Table 1 for explanation of abbreviations. Significant regressions are labeled by a continuous line.

M_2_ receptor density in the CPu of control rats (group C) exhibited a mean concentration of 949 ± 95 fmol/mg (mean ± SD) showing no significant side differences (Figures 2A,C). 6-OHDA lesioned animals (group L) demonstrated no significant interhemispheric differences (ipsilateral decrease of 6%) when the mean effect was calculated over the entire post injection time (Figures 2B,C). If this interhemispheric difference was analyzed along the post-injection time course, the right-left difference after 6-OHDA application was time-dependent (*R*^2^ = 0.12267, *p* = 0.025): −0.7% (L3W), 2.0% (L6W), −13.0% (L6W1M), −6.1% (L6W3M), −6.7% (L6W6M), −7.3% (L6W9M) (Figure 2D).

Striatal M_2_ agonist binding of rats treated only with BoNT-A (group B) showed no significant interhemispheric differences when the mean was calculated over all post-injection time points (Figure 2C). However, values changed significantly (*R*^2^ = 0.59512, *p* = 0.001) depending on post-injection times of BoNT-A starting with an ipsilateral decrease at 2 weeks and ending with an increase at 9 months: −8% (B2W), −13.8% (B1M), −0.4% (B3M), 2.7% (B6M), 13.3% (B9M) (Figure 2D).

M_2_ agonist binding was not significantly altered by post-treatment of hemi-PD rats with BoNT-A (group LB) or with vehicle (group LV) when the mean effect was calculated over all survival times (Figure 2C). Rats of the LB group showed an interhemispheric compensation of the density reductions caused by 6-OHDA injections which reduced the difference in the ipsilateral side over time (*R*^2^ = 0.19002, *p* = 0.010): (LB6W1M −8.9%, LB6W3M −6.9%, LB6W6M 0.4%, LB6W9M 2.2%) (Figure 2D).

We found no significant correlation (*R*^2^ = 0.2559, *p* = 0.0544) between the interhemispheric difference of these M_2_ receptor binding sites for groups LB and LV and the apomorphine-induced rotations.

### Muscarinic M_2_ and M_4_ receptor (antagonist binding)

**Figure 3:**
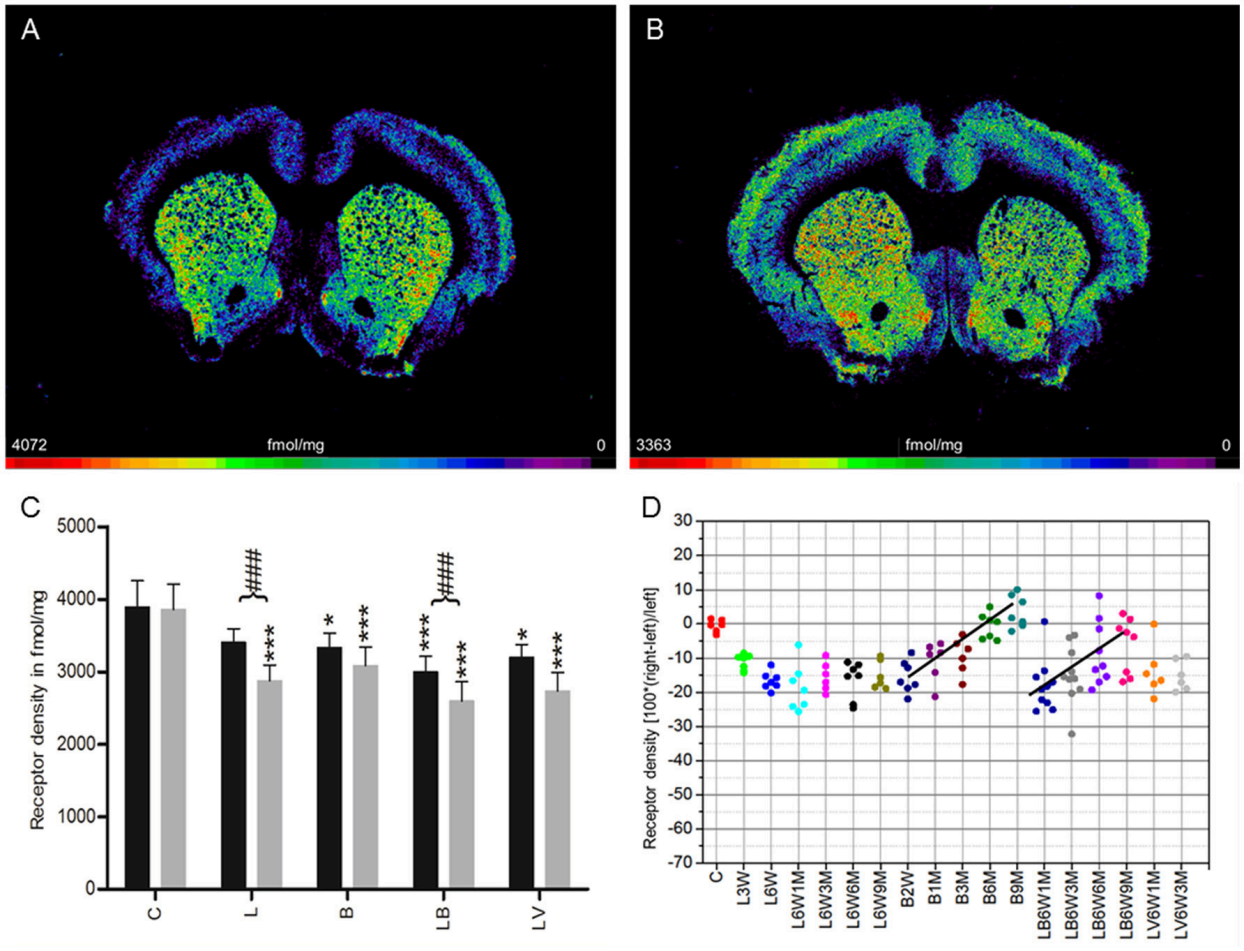
**(A,B)** Contrast-enhanced color-coded images showing the regional distribution of M_2_ and M_4_ receptor densities receptor density labeled with the antagonist [^3^H]AF-DX 384 in a control rat **(A,B)** after 6-OHDA lesion (group L6W6M) in a control rat **(A)** and after 6-OHDA lesion (group L6W6M) **(B)**. **(C)** Mean receptor density (fmol/mg protein; averaged over all post-lesion survival times) in the left/contralateral (black column) and right/ipsilateral hemispheres (gray column) of the five groups (see Figure 1). All data is expressed as means ± SD. Asterisks mark significant differences to the respective side of controls (^*^p < 0.05, ^***^p < 0.001). Rhombs signs highlight interhemispheric significance within each group (^###^*p* < 0.001). **(D)** Scatter plots and regression analyses of the right-left differences of M_2_ receptor density for all 18 groups and time points (five groups with different survival times); see Table 1 for explanation of abbreviations. Significant regressions are labeled by a continuous line.

In control rats (group C), a mean density of 3,876 ± 357 fmol/mg (mean ± SD) was measured. No significant interhemispheric differences were found (Figures 3A,C). Injection of 6-OHDA (group L) resulted in a significant ipsilateral decrease of antagonistic ([^3^H]-AFDX-384) binding of 16% when densities were averaged over all examined time points (Figures 3B–D). Furthermore, only densities of the ipsilateral hemisphere of group L animals were significantly lower than in controls. No significant correlation was found between receptor densities and post-injection time points (L6W1M −18.6%, L6W3M −15.4%, L6W6M −16.5%, L6W9M −15%) (Figure 3D).

Treatment with BoNT-A only (group B) resulted in a non-significant interhemispheric decrease (−8%) in antagonistic receptor binding when densities were averaged over all examined time points (Figure 3C). However, the densities were significantly lower in both hemispheres of group B rats compared to those of control animals (Figure 3C). Values changed significantly (*R*^2^ = 0.68926, *p* = 0.0001) depending on post BoNT-A injection-time, starting with an ipsilateral decrease at 2 weeks and ending with a slight increase at 9 months: −16% (B2W), −10.9% (B1M), −9.4% (B3M), −0.6% (B6M), 3.5% (B9M) (Figure 3D).

Group LB, but not group LV, showed a significant interhemispheric difference in receptor density if antagonistic binding sites were averaged over all examined time points (Figure 3C). Both groups presented significantly lower densities of antagonistic binding sites in both hemispheres if compared to controls (Figure 3C). A significant time-dependent BoNT-A-induced effect on the 6-OHDA induced binding site density (group LB) is disclosed in the longer post injection periods (*R*^2^ = 0.317, *p* = 0.0001): (LB6W1M −19.8%, LB6W3M −16.5%, LB6W6M −10.1%, LB6W9M −7.7% (Figure 3D). Contrastingly, the vehicle injection in hemi-PD rats (group LV) did not influence the 6-OHDA induced M_2_ receptor density (Figures 3C,D).

Right-left differences for groups LB and LV did not correlate (*R*^2^ = 0.1247, *p* = 0.1966) with motor behavior in the apomorphine rotation test.

### Muscarinic M_3_ receptor

**Figure 4:**
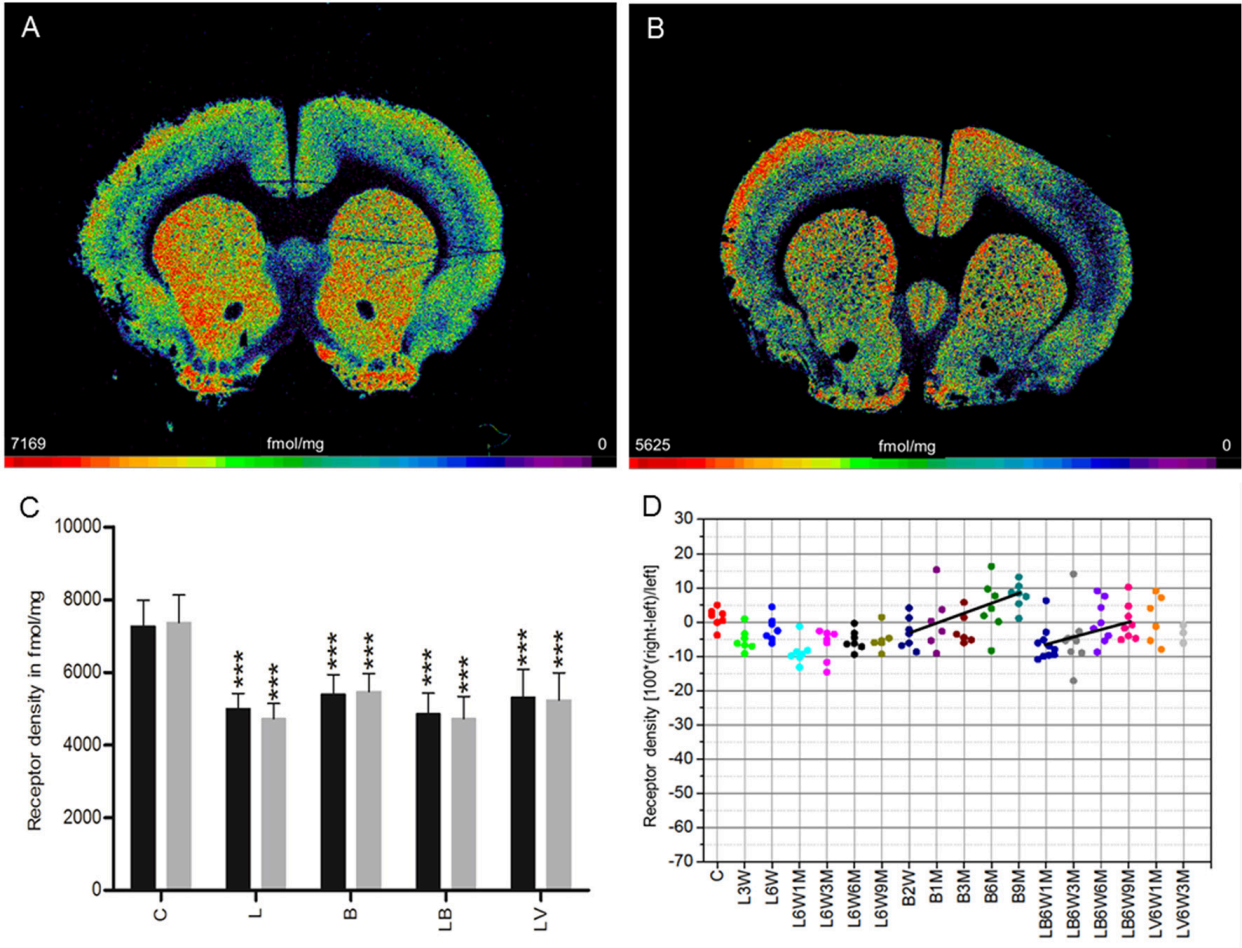
**(A,B)** Contrast-enhanced color-coded images showing the regional distribution of M_3_ receptor density labeled with [^3^H]4-DAMP in a control rat **(A,B)** after 6-OHDA lesion (group L6W1M) in a control rat **(A)** and after 6-OHDA lesion (group L6W1M) **(B)**. **(C)** Mean receptor density (fmol/mg protein; averaged over all post-lesion survival times) in the left/contralateral (black column) and right/ipsilateral hemispheres (gray column) of the five groups (see Figure 1). All data is expressed as means ± SD. Asterisks mark significant differences to the respective side of controls (^***^p < 0.001). **(D)** Scatter plots and regression analyses of the right-left differences of M_2_ receptor density for all 18 groups and time points (five groups with different survival times); see Table 1 for explanation of abbreviations. Significant regressions are labeled by a continuous line.

A mean (± SD) density of 7,321 ± 733 fmol/mg was found for M_3_ receptors in control rats (group C) (Figures 4A,C). The differences between both hemispheres are not significant in any of the examined control and experimental groups when values are averaged over all post-injection time points (Figures 4B,C). However, the M_3_ receptor density is significantly lower in both hemispheres of the experimental groups (L, B, LB, LV) compared to the drug naïve rats (group C) (Figure 4C).

A significant (*R*^2^ = 0.309, *p* = 0.001) time dependence of the BoNT-A effect (group B) on the M_3_ receptor densities was observed: −3% (B2W), –.3% (B1M), −2.0% (B3M), 4.4% (B6M), 7.8% (9M) (Figure 4D). The hemi-PD rats with injection of BoNT-A (group LB) also presented a significant (*R*^2^ = 0.16436, *p* = 0.020) post-injection time dependency (LB6W1M −5.6%, LB6W3M −5.1%, LB6W6M +0.6%, LB6W9M −1.4%) (Figure 4D). In the vehicle-injected group (LV), the M_3_ receptor density was comparable to that of hemi-PD rats (group L) (Figures 4C,D).

We observed no significant (*R*^2^ = 0.1433, *p* = 0.1643) correlation between the right-left difference of M_3_ receptor density in the CPu of groups LB and LV and the apomorphine-induced rotational behavior.

### Nicotinic α_4_β_2_ receptor

**Figure 5:**
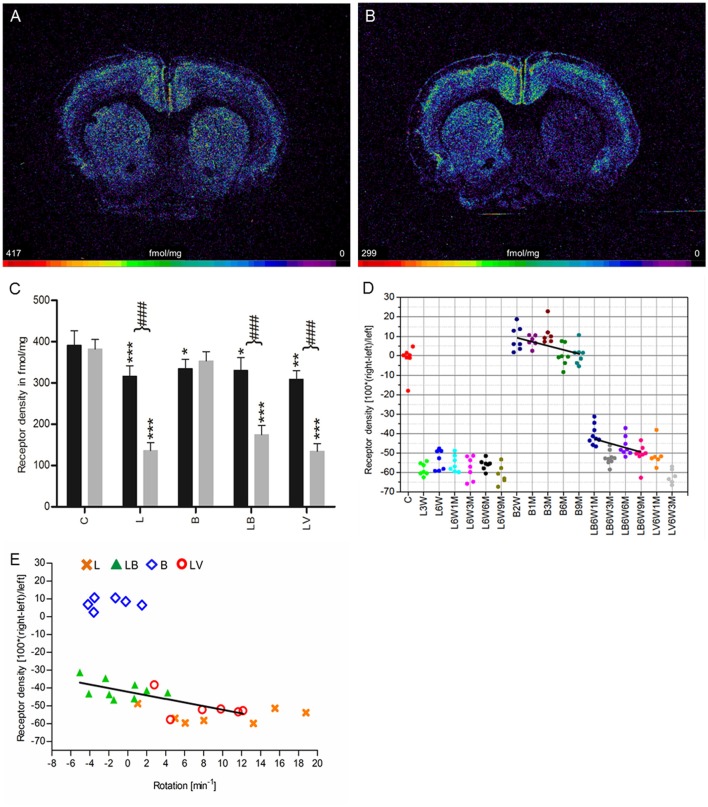
**(A,B)** Contrast-enhanced color-coded images showing the regional distribution of nicotinic α_4_β_2_ receptor density labeled with [^3^H]epibatidine in a control rat **(A,B)** after 6-OHDA lesion (group L6W3M) in a control rat **(A)** and after 6-OHDA lesion (group L6W3M) **(B)**. **(C)** Mean receptor density (fmol/mg protein; averaged over all post-lesion survival times) in the left/contralateral (black column) and right/ipsilateral hemispheres (gray column) of the five groups (see Figure 1). All data is expressed as means ± SD. Asterisks mark significant differences to the respective side of controls (^*^p < 0.05, ^**^p < 0.01, ^***^p < 0.001). Rhombs signs highlight interhemispheric significance within each group (^###^*p* < 0.001). **(D)** Scatter plots and regression analyses of the right-left differences of M_2_ receptor density for all 18 groups and time points (five groups with different survival times); see Table 1 for explanation of abbreviations. Significant regressions are labeled by a continuous line. **(E)** Relationship between interhemispheric differences in the α_4_β_2_ receptor densities of groups (LB and LV) and rotational behavior (anti-clockwise: +, clockwise: –).

Nicotinic α_4_β_2_ receptors exhibited a density of 387 ± 29 fmol/mg (mean ± SD) in the CPu of control rats (group C), and no significant interhemispheric differences were observed (Figures 5A,C). A significant reduction of 57% ipsilateral to the 6-OHDA application (group L) was found. The decrease remained constant up to 9 months after 6-OHDA injection (Figures 5B–D). Furthermore, this obvious interhemispheric difference was also seen in the hemi-PD rats treated with BoNT-A (group LB) or vehicle (group LV). A significant, but low reduction in binding site densities is also found on the contralateral side of all experimental groups (L, B, LB, LV) if compared to drug naïve controls (Figure 5C).

Treatment with BoNT-A only (group B) did not result in significant interhemispheric differences when densities were averaged over all post-injection time points (Figure 5C), although the receptor density of the ipsilateral hemisphere decreased significantly (*R*^2^ = 0.2678, *p* = 0.002) from values well above control levels at 2 weeks to 3 months after BoNT-A to control values at 6 and 9 months post-injection (B2W 9.0%, B1M 7.6%, B3M 11.4%, B6M 0.1%, B9M 0.7%) (Figure 5D).

If the time-dependency after BoNT-A injection was analyzed (group LB, Figure 5D), a significant (R^2^ = 0.174, p = 0.014) negative correlation between interhemispheric differences and increasing post-injection times was found (LB6W1M −41.8%, LB6W3M −53.4%, LB6W6M −46.2%, LB6W9M −51.8%) (Figure 5D). In vehicle-injected hemi-PD rats (LV) nicotinic receptors equaled those of group L (Figures 5C,D).

A highly significant correlation (*R*^2^ = 0.5493, *p* = 0.0016) of the interhemispheric difference of nicotinic receptors in the groups LB and LV and apomorphine-induced rotations was found (Figure 5E).

### Summary of important results

Unilateral injection of 6-OHDA into the MFB (group L) leads to a 10% decrease of M_1_ receptor densities 3 weeks after 6-OHDA application, and a restitution to control values 9 months later (Figure 1D). The M_2_/M_4_ receptors (agonistic binding site) show control values 3 weeks after 6-OHDA application followed by a decrease of ~10% over the subsequent 9 months (Figure 2D). The M_2_ receptor (antagonistic binding site) and the M_3_ receptor are also decreased by ~10% compared to control value, but do not show any significant recovery over time (Figures 3D, 4D). The nicotinic α_4_β_2_ receptor shows the most obvious decrease in density (50–60%) 3 weeks after 6-OHDA injection, but no sign of any recovery during the following period (Figure 5D).

Injection of BoNT-A into the striatum of drug naïve rats (group B) leads to an initial (2 weeks after injection) decrease in M_1_ and M_3_ receptor densities of about 5–10% followed by a steady recovery, reaching 5–10% over the control value 9 months later (Figures 1D, 4D). Also the M_2_ receptor (agonistic binding site) show an initial decrease of slightly more than 10%, followed by a steep increase resulting in a density of ~10% over control values 9 months after BoNT-A treatment (Figure 2D). The antagonistic binding sites of the M_2_/M_4_ receptor start with a density nearly 20% below control level, and come back to control levels 9 months later (Figure 3D). The nicotinic α_4_β_2_ receptor shows a completely different reaction. It starts with a 10% higher density than control values 2 weeks after BoNT-A injection, and returns to control values during the following period (Figure 5D).

Injection of BoNT-A into the 6-OHDA lesioned striatum (group LB) leads to an M_1_ receptor density which is well comparable to that of the untreated (group L) and vehicle injected (group LV) hemi-PD rats over all time points (Figure 1D). The decrease in M_2_ (agonist and antagonist binding sites) and M_3_ receptor densities caused by 6-OHDA application is compensated by BoNT-A, since changes over time in the densities of these receptors in the LB group result in values comparable to those of controls 9 months after BoNT-A injection (Figures 2D, 4D). The nicotinic α_4_β_2_ receptor shows an initial 15% increase of its density when groups L and LB are compared. However, the receptor density decreases during the following period and in the hemiPD rats treated with BoNT-A (LB group) reaches nearly the values of the untreated hemi-PD (L group) rats (Figure 5D). The large difference between the non-lesioned but BoNT-A treated rats (group B) and the BoNT-A treated hemi-PD rats (group LB) is remarkable and may indicate a strong effect of 6-OHDA lesions on the expression of the nicotinic α_4_β_2_ receptor. This is also supported by the conspicuously large decrease of the α_4_β_2_ receptors after 6-OHDA lesioning.

Most notable is the fact that BoNT-A injection causes a negative correlation between nicotinic α_4_β_2_ receptor densities and post-injection time in drug naïve (group B) and BoNT-A treated (group LB) hemi-PD rats, but results in a positive correlation between all examined muscarinic cholinergic binding site (M_1_, M_2_/M_4_ (agonist and antagonist) and M_3_) densities. Apparently, the nicotinic and muscarinic receptors react differentially to BoNT-A treatment.

## Discussion

Progressive dopaminergic cell death and the consequent DA deficit in the CPu can only partly explain PD's symptomatology, hence various compensatory changes in dopaminergic and other neurotransmitter systems have been described (Barone, 2010). Here, we applied quantitative *in vitro* receptor autoradiography to analyze time-dependent changes in the densities of mAchRs (M_1_, M_2_, M_3_, M_4_) and nAchRs (α_4_β_2_) in hemi-PD rats with or without unilateral BoNT-A injection into the striatum. Receptor densities were measured in the striatum ipsi- and contralateral to the 6-OHDA injection side, and expressed as relative values by calculating the difference between ipsilateral and contralateral receptor densities normalized by the contralateral density. BoNT-A was injected ipsilaterally.

Five experimental groups were analyzed up to a survival time of 9 months to uncover changes in hemi-PD rat striata and to disclose possible receptor-related explanations for the positive motor effect of BoNT-A in hemi-PD rats (Wree et al., 2011; Antipova et al., 2013; Mann et al., 2018a,b). To confirm successful 6-OHDA lesion we performed apomorphine-induced rotational testing. As dopaminergic deprivation with 6-OHDA increased ipsilateral striatal D_2_/D_3_ receptor density (Creese et al., 1977; Creese and Snyder, 1979; Murrin et al., 1979; Staunton et al., 1981; Przedbroski et al., 1995; Sun et al., 2011; Choi et al., 2012), the ipsilateral CPu of hemi-PD rats is inhibited more than the left by application of the D_2_/D_3_ agonist apomorphine. Hemi-PD rats start to rotate contralateral to the 6-OHDA lesion. Animals which demonstrated at least 4 rotations per minute were seen as successfully lesioned confirming dopaminergic depletion of about 97% (Ungerstedt and Arbuthnott, 1970).

### Striatal organization and cholinergic neurotransmission

The majority of striatal neurons (about 95%; Oorschot, 1996) are medium spiny cells (MSN), a GABAergic projection neuron. About 50% express substance P, dynorphin and dopamine D_1_ receptors, and project to the globus pallidus internus and substantia nigra pars reticulata (SNpr) (direct basal ganglia loop), whereas the other half of the MSNs express enkephalin as well as dopamine D_2_ receptors and mainly project to the globus pallidus externus (EGP) (indirect basal ganglia loop; Bolam et al., 2000). The remaining 3–5% are interneurons and can be subdivided into at least 4 subtypes. Three of them are GABAergic interneurons expressing either parvalbumin, somatostatin or calretinin. The fourth subtype is a large tonically active aspiny cholinergic interneuron (Kawaguchi, 1993; Kawaguchi et al., 1995). Cholinergic neurotransmission in the CPu arises predominantly from these cholinergic interneurons, but also from an external cholinergic projection from the pedunculopontine and laterodorsal tegmental area (Woolf and Butcher, 1986; Dautan et al., 2014). Moreover, a weak cholinergic input was described to come from the nucleus basalis Meynert (Mesulam, 2013).

### Localization of cholinergic receptors

In the CPu, M_1_ receptors are expressed on MSNs (Araujo et al., 1991; Hersch et al., 1994; Yung et al., 1995; Muccioli et al., 1996; Alcantara et al., 2001; Yan et al., 2001; Bauer et al., 2005; Haghir et al., 2009). M_2_ receptors are also located on MSNs and ChAT-ir interneurons (Mash and Potter, 1986; Hersch et al., 1994; Bernard et al., 1998; Bauer et al., 2005; Haghir et al., 2009). M_3_ receptors are found on MSNs and on terminals of cortical and thalamic afferents (Zubieta and Frey, 1993; Levey et al., 1994; Yan et al., 2001; Haghir et al., 2009). M_4_ receptors are found in spines of MSNs of striatal neurons (Levey et al., 1991; Hersch et al., 1994). Within the CPu, nAchR are described predominantly on nigrostriatal and also corticostriatal terminals, and ChAT-ir interneurons (Hunt and Schmidt, 1978; Schwartz and Kellar, 1983; Clarke et al., 1985; Happe et al., 1994; Arroyo-Jim nez et al., 1999; Kaiser and Wonnacott, 2000; Jones et al., 2001; Pradhan et al., 2002).

### Cholinergic and dopaminergic interaction

The dopaminergic and cholinergic systems can regulate one another's function in a bidirectional manner (Havekes et al., 2011). Receptors play a key role in this issue. Activation of dopamine D_2_ receptors inhibits Ach release (Maurice et al., 2004), whereas the D_1_ receptors facilitate it (Joyce, 1991). The nAchRs and mAchRs (M_1_−M_5_) were both described to mediate DA release from dopaminergic terminals. However, studies report conflicting results on the DA inhibiting or facilitating effects. The role of different receptor subtypes, especially of mAchRs located on MSNs and corticostriatal or thalamostriatal afferents (Woolf and Butcher, 1981; Schoffelmeer et al., 1986; Xu et al., 1989; De Klippel et al., 1993; Calabresi et al., 2000; Zhang et al., 2002; Bendor et al., 2010; Havekes et al., 2011) is not completely understood. NAchRs located on dopaminergic axons were described to regulate DA release (Sharples et al., 2000; Wonnacott et al., 2000; Zhou et al., 2001). Moreover, due to the regulation of Ach release of the cholinergic interneurons via autoreceptors (M_2_ + M_4_), the DA release of dopaminergic terminals is modified indirectly by mAchRs via nAchRs (Raiteri et al., 1984). A recent publication postulated a change in the impact of Ach on the regulation of DA release (reducing or increasing) at DA terminals depending on the firing mode of the respective DA neuron (Threlfell et al., 2010). DA neurons exhibit two different states of firing modes in rats *in vivo*: a single-spike mode at a frequency of about 1–10 Hz, and a burst firing mode of 3–5 spikes at a frequency of about 15–100 Hz which is interrupted by short-term pauses (Hyland et al., 2002). Ach acting on β_2_ subunits of nAchRs on nigrostriatal terminals provokes DA release in terminals during the single-spike mode, but inhibits DA release at terminals in the burst firing mode (Zhou et al., 2001; Rice and Cragg, 2004; Zhang and Sulzer, 2004). Activation of mAchRs (M_2_, M_4_) results in decreased Ach release from cholinergic interneurons, and by this in a reduced activation of nAchR on dopaminergic axons. Consequently, inhibition of DA release is observed after activation of mAchRs for single-spike modes of DA neurons, activation is found for burst firing mode (Threlfell et al., 2010).

### Muscarinic receptors in PD and hemi-PD

Studies on mAchR binding in the CPu of PD patients using post-mortem brain tissue (Aubert et al., 1992; Lange et al., 1993; McOmish et al., 2017) are partly contradictory and have been summarized together with animal experiments in Table 4. Lange et al. (1993) postulated a decrease in M_1_ receptor density of the striatum and an unaltered M_2_ receptor density in PD brain tissue compared to controls (Lange et al., 1993). McOmish et al. (2017) recently found no changes in M_1_ and M_2_ receptor densities, but an increased M_3_ receptor density in the CPu of PD patients (McOmish et al., 2017).

**Table 4:** Striatal changes in cholinergic receptor densities in human PD patients and different animal models of PD.

**Receptor subtype**	**Species/Animal model**	**Survival time**	**Detection method**	**Ligand**	**Effects**	**References**
M_1_	Human PD patient	–	Binding in post-mortem homogenates	[^3^H]pirenzepine	Decrease	Lange et al., 1993
M_1_	Human PD patient	–	Binding in post-mortem slices	[^3^H]pirenzepine	Unaltered	McOmish et al., 2017
M_1_	MFB-lesioned 6-OHDA rat	1 year	Autoradiography	[^3^H]pirenzepine	Decrease (26%)	Dawson et al., 1991
M_1_	SN-lesioned 6-OHDA rat	3 weeks	Autoradiography	[^3^H]pirenzepine	Decrease (25–29%)	Joyce, 1991
M_1_	MFB-lesioned 6-OHDA rat	3 weeks	Autoradiography	[^3^H]pirenzepine	Unaltered	Wang et al., 2014
M_1_	MFB-lesioned 6-OHDA rat	10-13 days	SPECT	[^123^I]iododexetimide	Unaltered	Knol et al., 2014
M_2_	Human PD patients	–	Binding in post-mortem homogenates	[^3^H]oxotremorine-M	Unaltered	Lange et al., 1993
M_2_	Human PD patients	–	Binding in post-mortem slices	[^3^H]AF-DX 384	Unaltered	McOmish et al., 2017
M_2_	SN-lesioned 6-OHDA rat	3 weeks	Autoradiography	[^3^H]NMS (+unlabeled pirenzipine)	Decrease (27%)	Joyce, 1991
M_2_	Pitx3ak mice	4–6 months	Autoradiography	[^3^H]oxotremorine-M	Decrease (19%)	Cremer et al., 2015
M_3_	Human PD patients	–	Binding in post-mortem slices	[^3^H]4DAMP	Increase	McOmish et al., 2017
M_3_	Pitx3ak mice	4–6 months	Autoradiography	[^3^H]4DAMP	Unaltered	Cremer et al., 2015
M_1_-M_5_	MFB-lesioned 6-OHDA rat	8 weeks	Autoradiography	[^3^H]QNB	Decrease (12–17%)	Araki et al., 2000
nAch	Human PD patients	–	Binding in post-mortem homogenates	[^3^H]nicotine	Unaltered	Lange et al., 1993
nAch	Human PD patients	–	Binding in post-mortem slices	[^3^H]methylcarbamylcholine	Decrease (74%)	Aubert et al., 1992
nAch	Human PD patients	–	PET	2-[^18^F]FA-85380	Decrease (20%)	Meyer et al., 2009
nAch	MFB-lesioned 6-OHDA rat	2 weeks	Ligand-binding techniques	[^3^H]epibatidine	Decrease (50%)	Zoli et al., 2002
nAch	MPTP-treated monkeys	4 weeks	Autoradiography	[^123^I]epibatidine	Decrease (40-50%)	Kulak et al., 2002
nAch	MFB-lesioned 6-OHDA rat	3 weeks	Autoradiography	[^125^I]epibatidine	Decrease (36%)	Pradhan et al., 2002
nAch	MFB-lesioned 6-OHDA rat	4–5 weeks	Autoradiography	[^125^I]epibatidine	Decrease (47%)	Perez et al., 2010
nAch	Pitx3ak mice	4–6 months	Autoradiography	[^3^H]epibatidine	Decrease (47%)	Cremer et al., 2015

*The table summarizes the main studies of this field*.

M_2_ receptors contain a high and a low affinity binding site (Birdsall et al., 1978; Giraldo et al., 1987). Here, we investigated the high affinity binding site of the M_2_ receptor with the agonist [^3^H]oxotremorine-M and the high and low affinity binding site with the antagonist [^3^H]AF-DX 384. Furthermore, it must be noted that [^3^H]AF-DX 384 also binds to the antagonistic site of the M_4_ receptor, and about 80% of the sites labeled with this ligand in the mouse striatum correspond to the M_4_ and not the M_2_ receptor (Levey et al., 1991; Hersch et al., 1994; Valuskova et al., 2018). Consequently, we found an about four times higher mean density with [^3^H]AF-DX 384 than with the more M_2_ subtype-specific [^3^H]oxotremorine-M throughout all experimental groups (Figures 2C, 3C, Table 3). This is in line with the measurement of Svensson et al. (1992) who described that the high affinity binding sites constitute about 27% and the low affinity site about 73% (Svensson et al., 1992). Interestingly, only the [^3^H]AF-DX 384 binding significantly decreases after 6-OHDA lesioning (Figure 3C), and slowly returns to control values after BoNT-A injection (Figure 3D). With the presently available tritiated ligands it cannot be clarified whether this decrease is caused by the higher binding of M_4_ than M_2_ receptors.

In hemi-PD rats, all mAchR densities were decreased ipsilateral to the lesion between 6% and 16% as compared to the contralateral hemisphere. Our findings on 6-OHDA-induced changes of mAchRs are partly in line with others: Araki et al. (2000) showed that dopaminergic depletion by 6-OHDA injection into the MFB decreased binding of [^3^H]QNB (labeling subtypes M_1_-M_5_; Jakubík et al., 2017) by 12–17% 8 weeks post lesion (Araki et al., 2000). Similar experiments resulted in a striatal decrease of M_1_ receptor density of 26% using the selective antagonist [^3^H]pirenzepine about 1 year after deafferentiation (Dawson et al., 1991), and also a decrease in [^3^H]pirenzepine binding of 25% to 29% after intranigral 6-OHDA application (Joyce, 1991). However, contradictory results were published by Wang et al. (2014) who found no changes in [^3^H]pirenzepine binding 3 weeks post 6-OHDA lesion of the MFB (Wang et al., 2014) as well as by Knol et al. (2014) describing unaltered striatal M_1_ receptor density with the [^123^I]iododexetimide SPECT method. Besides the small general reduction of the striatal M_1_ receptor density ipsilateral to the 6-OHDA lesion, we found a positive correlation between the right-left difference and the survival time between 3 weeks and 9 months. M_2_ receptors measured using [^3^H]NMS in the presence of unlabeled pirenzepine decreased by about 27% in the lateral CPu after intranigral 6-OHDA injection (Joyce, 1991). Also in Pitx3ak mice, which exhibit a severe loss of dopaminergic cells in the SNpc, M_2_ receptor density (agonist binding) in the CPu was significantly reduced by 19% (Cremer et al., 2015). In addition to the initial marked reduction of M_2_ receptor density induced by dopaminergic deafferentiation, the M_2_ receptor densities further significantly decreased with longer post lesion survival times up to 9 months in the present study. We found significant reductions of M_3_ receptor densities, notably in both hemispheres, after 6-OHDA lesion. This is in contrast to results in the CPu of DA depleted Pitx3ak mice, where Cremer et al. (2015) found no significant change in M_3_ receptor densities as compared to wildtype mice. This contradiction may be explainable by the completely different PD models in the work of Cremer et al. (2015) and the present study.

When inducing hemi-PD by 6-OHDA injection, we did not use desipramine application ahead (Mailman, 1983; López-Giménez et al., 1997; Nash and Brotchie, 2002) in order to avoid binding of this substance to various receptors, and thus to ensure comparability between all experimental groups including BoNT-A treatment (Wree et al., 2011; Antipova et al., 2013; Hawlitschka et al., 2013; Mehlan et al., 2016). Notably, lesion of the locus coeruleus did not cause changes in mAchR densities using [^3^H]QNB binding (Sharma et al., 1981). Moreover, striatal Ach levels and M_1_ receptor density did not show any changes after specific noradrenergic depletion with the selective noradrenergic neurotoxin N-2-chloroethyl-N-ethyl-2-bromobenzylamine (DSP-4) (Asanuma et al., 1992). Following dopaminergic depletion the densities of all mAchRs were found to be reduced in the present study. As there is no evidence in recent literature for the localization of mAchRs on dopaminergic axon terminals (Jones et al., 2001; Zhou et al., 2001), the reduction of mAchR densities can probably not be explained by 6-OHDA-induced axonal degeneration. Rather, the hyperactivity of striatal cholinergic neurotransmission due to dopaminergic denervation of the CPu and the subsequently increased Ach levels in hemi-PD rats (DeBoer et al., 1993; Muma et al., 2001; Rakovska et al., 2003) might result in a downregulation of mAchRs. This assumption is in line with findings that chronic inhibition of acetylcholinesterase inducing increased Ach concentrations also leads to a significant decrease of about 20% in mAchR densities as revealed by [^3^H]QNB binding (Sivam et al., 1983; Yamada et al., 1983).

### Muscarinic receptors in BoNT-A-injected rats

BoNT-A injection in control animals always led to an interhemispheric right-left difference of mAchRs between −7 and −16% 2 weeks after injection that significantly diminishes over time and even resulted in an ipsilateral increase in receptor densities between 3.5 and 13.3% 9 months later. BoNT-A injection in hemi-PD rats significantly affected overall interhemispheric differences for M_2_/M_4_ (antagonist binding) and M_3_ receptors, whereas differences of M_1_, and M_2_ (agonist binding) receptors were not significantly changed (Figures 1C, 4C). However, considering the time course of the BoNT-A effect in hemi-PD rats, interhemispheric differences in mAchR densities diminished with increasing survival time. The effect of BoNT-A on mAchR densities found in hemi-PD rats, however, did not correlate with changes in apomorphine-induced rotations. Seemingly, the positive effect of BoNT-A on motor behavior in hemi-PD rats for up to 6 months (Wree et al., 2011) cannot be based on changes in mAchR densities.

### Nicotinic receptors in PD and hemi-PD

In PD patients striatal nAchRs levels were analyzed with conflicting results (see Table 4 for a summary): Aubert et al. (1992) using *in vitro* receptor autoradiography reported a decrease by 74%, and Meyer et al. (2009) using PET analysis with 2-[^18^F]FA-85380 found a reduction of 20%. However, Lange et al. (1993) evaluating *in vitro* receptor binding studies did not find changes in the caudate nucleus or in the putamen. In the CPu of MPTP-treated squirrel monkeys nAchRs density was also reported to decline by 40% to 50% using [^3^H]epibatidine (Kulak et al., 2002).

Hemi-PD rats showed a massive ipsilateral decrease in striatal nAchRs of more than 50%. This effect was constant up to a survival time of 9 months. This confirms previous studies: Zoli et al. (2002) demonstrated an ipsilateral decrease of 50% in nAchR density using [^3^H]epibatidine 2 weeks after 6-OHDA, Pradhan et al. (2002) reported a decrease of about 36% of [^125^I]epibatidine binding 3 weeks post 6-OHDA, and Perez et al. (2010) an ipsilateral decrease of 47%. Vehicle injections did not cause any nAchR changes in the CPu (Pradhan et al., 2002). The lesion studies in rats are in line with findings in the dopamine-depleted Pitx3ak mouse, where striatal nAchR density was also drastically reduced by 47% relative to control animals (Cremer et al., 2015).

This dramatic loss of nAchR density after destruction of dopaminergic SNpc neurons may be explained by the predominant localization of nAchRs on dopaminergic axonal terminals (Kaiser and Wonnacott, 2000; Jones et al., 2001; Pradhan et al., 2002). About 50% of the striatal nAchRs are located on cortical afferents or striatal interneurons (Zoli et al., 2002), thus explaining the remaining striatal [^3^H]epibatidine binding after 6-OHDA lesion.

### Nicotinic receptors in BoNT-A-injected rats

NAchRs were slightly increased ipsilateral one month after BoNT-A injection. However, a time dependence was detected: this initial increase was followed thereafter by a normalization to control values. In BoNT-A injected hemi-PD rats the interhemispheric difference of nAchRs density significantly diminished by about 10% compared to both the vehicle-injected rats (group LV) and the drug naïve hemi-PD rats (group L). Thus, BoNT-A induced an increase of nAchR density in groups B and LB.

Seemingly, BoNT-A induced an increase of nAchRs on non-dopaminergic structures, e.g., cortical afferents or striatal interneurons, since dopaminergic terminals no longer exist in hemi-PD. An initially increased nAchR density compared to controls and vehicle-injected rats was found after BoNT-A injection which may be explained by the reduction of striatal Ach content and a concomitant upregulation of nAchRs. This effect, however, disappears after 6–9 months after BoNT-A injection. Interestingly, the opposite effect of BoNT-A was seen in mAchR densities; here the reduction in striatal Ach content was followed by a downregulation of mAchR densities.

This study expands our understanding of 6-OHDA- and BoNT-A-induced changes in striatal mAchR and nAchR expression. It provides hypotheses for the positive BoNT-A effect on motor performance in hemi-PD rats based on changes in cholinergic receptor densities. The differential outcome of striatal BoNT-A application on Ach receptor densities can be interpreted as a further hint to the fact, that the densities of mAchRs and nAchRs are regulated differently by the availability of its transmitter (Sivam et al., 1983; Hefco et al., 2004). Quantification of the receptor densities of mAchRs and nAchRs provides evidence that cholinergic transmission has a significant impact on the clinical symptoms associated with DA depletion. Also other neurotransmitter receptors, especially glutamatergic and GABAergic receptors on cortical and thalamic terminals are of interest. Changes in corticostriatal glutamate release in hemi-PD rats have already been reported (Lindefors and Ungerstedt, 1990). To comprehensively characterize the receptor-mediated BoNT-A effect, the reaction not only of dopamine, noradrenaline, and serotonin receptors (Mann et al., 2018b; Wedekind et al., 2018), but also of glutamate and GABA receptors should be investigated.

## Data availability statement

The raw data supporting the conclusions of this manuscript will be made available by the authors, without undue reservation, to any qualified researcher.

## Author contributions

AW and KZ designed and planned the project. AW performed 6-OHDA lesioning and BoNT-A injections. OS and AW provided the analyzing strategy, analyzed data, and designed concepts for figures. AH performed apomorphine-induced rotation tests and MC the receptor binding experiments. FK and TM analyzed data. TM, AW, NP-G, and KZ interpreted data and wrote the manuscript. All authors have approved the final article.

### Conflict of interest statement

The authors declare that the research was conducted in the absence of any commercial or financial relationships that could be construed as a potential conflict of interest.

## Abbreviations

6-OHDA: 6-hydroxydopamine
Ach: acetylcholine
BoNT-A: botulinum neurotoxin-A
BSA: bovine serum albumin
CPu: caudate-putamen
DA: dopamine
DSP-4: N-2-chloroethyl-N-ethyl-2-bromobenzylamine
EGP: externus globus pallidus
hemi-PD: hemiparkinsonian
M_1_: acetylcholine muscarinic M_1_ receptor
M_2_: acetylcholine muscarinic M_2_ receptor
M_3_: acetylcholine muscarinic M_3_ receptor
MFB: medial forebrain bundle
MSN: striatal medium spiny neuron
mAchRs: muscarinic acetylcholine receptors
nAchRs: nicotinic acetylcholine receptors
PBS, phosphate-buffered saline, PD: Parkinson's disease
ROI: region of interest
SNpc: substantia nigra pars compacta
SNpr, substantia nigra pars reticulate, SNAP25: synaptosome-associated glycoprotein of 25-kDa.

## References

[B1] Alcantara A. A. Mrzljak L. Jakab R. L. Levey A. I. Hersch S. M. Goldman-Rakic P. S. (2001). Muscarinic m1 and m2 receptor proteins in local circuit and projection neurons of the primate striatum: anatomical evidence for cholinergic modulation of glutamatergic prefronto-striatal pathways. J. Comp. Neurol. 434, 445–460. 10.1002/cne.1186 11343292

[B2] Antipova V. A. Holzmann C. Schmitt O. Wree A. Hawlitschka A. (2017). Botulinum neurotoxin a injected ipsilaterally or contralaterally into the striatum in the Rat 6-OHDA model of unilateral parkinson's disease differently affects behavior. Front. Behav. Neurosci. 11:119. 10.3389/fnbeh.2017.00119 28680396 PMC5478737

[B3] Antipova V. Hawlitschka A. Mix E. Schmitt O. Dräger D. Benecke R.. (2013). Behavioral and structural effects of unilateral intrastriatal injections of botulinum neurotoxin a in the rat model of Parkinson's disease. J. Neurosci. Res. 91, 838–847. 10.1002/jnr.23210 23553727

[B4] Aosaki T. Miura M. Suzuki T. Nishimura K. Masuda M. (2010). Acetylcholine-dopamine balance hypothesis in the striatum: an update. Geriatr. Gerontol. Int. 10(Suppl. 1), 148–157. 10.1111/j.1447-0594.2010.00588.x 20590830

[B5] Araki T. Tanji H. Fujihara K. Kato H. Imai Y. Mizugaki M.. (2000). Sequential changes of cholinergic and dopaminergic receptors in brains after 6-hydroxydopamine lesions of the medial forebrain bundle in rats. J. Neural Transm. 107, 873–884. 10.1007/s007020070039 11041269

[B6] Araujo D. M. Lapchak P. A. Quirion R. (1991). Heterogeneous binding of [3H]4 DAMP to muscarinic cholinergic sites in the rat brain: evidence from membrane binding and autoradiographic studies. Synapse 9, 165–176. 10.1002/syn.890090303 1776129

[B7] Arroyo-Jim nez M. M. Bourgeois J. P. Marubio L. M. Le Sourd A. M. Ottersen O. P. Rinvik E.. (1999). Ultrastructural localization of the alpha4-subunit of the neuronal acetylcholine nicotinic receptor in the rat substantia nigra. J. Neurosci. 19, 6475–6487. 10414976 10.1523/JNEUROSCI.19-15-06475.1999 PMC6782828

[B8] Asanuma M. Ogawa N. Haba K. Hirata H. Mori A. (1992). Effects of chronic catecholamine depletions on muscarinic M1-receptor and its mRNA in rat brain. J. Neurol. Sci. 110, 205–214. 1506860 10.1016/0022-510x(92)90029-k

[B9] Aubert I. Araujo D. M. Cécyre D. Robitaille Y. Gauthier S. Quirion R. (1992). Comparative alterations of nicotinic and muscarinic binding sites in Alzheimer's and Parkinson's diseases. J. Neurochem. 58, 529–541. 1729398 10.1111/j.1471-4159.1992.tb09752.x

[B10] Barone P. (2010). Neurotransmission in Parkinson's disease: beyond dopamine. Eur. J. Neurol. 17, 364–376. 10.1111/j.1468-1331.2009.02900.x 20050885

[B11] Bauer A. Zilles K. Matusch A. Holzmann C. Riess O. Von Hörsten S. (2005). Regional and subtype selective changes of neurotransmitter receptor density in a rat transgenic for the Huntington's disease mutation. J. Neurochem. 94, 639–650. 10.1111/j.1471-4159.2005.03169.x 16033418

[B12] Benarroch E. E. (2012). Effects of acetylcholine in the striatum. Recent insights and therapeutic implications. Neurology 79, 274–281. 10.1212/wnl.0b013e31825fe154 22802594

[B13] Bendor J. Lizardi-Ortiz J. E. Westphalen R. I. Brandstetter M. Hemmings H. C. Sulzer D.. (2010). AGAP1/AP-3-dependent endocytic recycling of M5 muscarinic receptors promotes dopamine release. EMBO J. 29, 2813–2826. 10.1038/emboj.2010.154 20664521 PMC2924642

[B14] Bernard V. Laribi O. Levey A. I. Bloch B. (1998). Subcellular redistribution of m2 muscarinic acetylcholine receptors in striatal interneurons *in vivo* after acute cholinergic stimulation. J. Neurosci. 18, 10207–10218. 9822774 10.1523/JNEUROSCI.18-23-10207.1998 PMC6793283

[B15] Bernheimer H. Birkmayer W. Hornykiewicz O. Jellinger K. Seitelberger F. (1973). Brain dopamine and the syndromes of Parkinson and Huntington. Clinical, morphological and neurochemical correlations. J. Neurol. Sci. 20, 415–455. 4272516 10.1016/0022-510x(73)90175-5

[B16] Birdsall N. J. Burgen A. S. Hulme E. C. (1978). The binding of agonists to brain muscarinic receptors. Mol. Pharmacol. 14, 723–736. 714021

[B17] Blandini F. Nappi G. Tassorelli C. Martignoni E. (2000). Functional changes of the basal ganglia circuitry in Parkinson's disease. Prog. Neurobiol. 62, 63–88. 10.1016/S0301-0082(99)00067-2 10821982

[B18] Bolam J. P. Hanley J. J. Booth P. A. Bevan M. D. (2000). Synaptic organisation of the basal ganglia. J. Anat. 196(Pt. 4), 527–542. 10.1046/j.1469-7580.2000.19640527.x 10923985 PMC1468095

[B19] Brooks D. J. (1998). The early diagnosis of Parkinson's disease. Ann. Neurol. 44(3Suppl. 1), S10–S18. 9749569 10.1002/ana.410440704

[B20] Calabresi P. Centonze D. Gubellini P. Pisani A. Bernardi G. (1998). Blockade of M2-like muscarinic receptors enhances long-term potentiation at corticostriatal synapses. Eur. J. Neurosci. 10, 3020–3023. 9758172 10.1111/j.1460-9568.1998.00348.x

[B21] Calabresi P. Centonze D. Gubellini P. Pisani A. Bernardi G. (2000). Acetylcholine-mediated modulation of striatal function. Trends Neurosci. 23, 120–126. 10.1016/S0166-2236(99)01501-5 10675916

[B22] Caleo M. Antonucci F. Restani L. Mazzocchio R. (2009). A reappraisal of the central effects of botulinum neurotoxin type A: by what mechanism? J. Neurochem. 109, 15–24. 10.1111/j.1471-4159.2009.05887.x 19154335

[B23] Carlsson A. Lindqyist M. Magnusson T. (1957). 3,4-Dihydroxyphenylalanine and 5-hydroxytryptophan as reserpine antagonists. Nature 180:1200. 13483658 10.1038/1801200a0

[B24] Choi J. Y. Kim C. H. Jeon T. J. Cho W. G. Lee J. S. Lee S. J. (2012). Evaluation of dopamine transporters and D2 receptors in hemiparkinsonian rat brains *in vivo* using consecutive PET scans of [18F]FPCIT and [18F]fallypride. Appl. Radiat. Isot. 70, 2689–2694. 10.1016/j.apradiso.2012.08.005 23041777

[B25] Clarke P. B. Schwartz R. D. Paul S. M. Pert C. B. Pert A. (1985). Nicotinic binding in rat brain: autoradiographic comparison of [3H]acetylcholine, [3H]nicotine, and [125I]-alpha-bungarotoxin. J. Neurosci. 5, 1307–1315. 10.1523/JNEUROSCI.05-05-01307.1985 3998824 PMC6565049

[B26] Coffield J. A. Considine R. V. Simpson L. L. (1994). Clostridial neurotoxins in the age of molecular medicine. Trends Microbiol. 2, 67–69. discussion: 69–72. 10.1016/0966-842X(94)90532-0 7908844

[B27] Cragg S. J. (2006). Meaningful silences: how dopamine listens to the ACh pause. Trends Neurosci. 29, 125–131. 10.1016/j.tins.2006.01.003 16443285

[B28] Creese I. Burt D. R. Snyder S. H. (1977). Dopamine receptor binding enhancement accompanies lesion-induced behavioral supersensitivity. Science 197, 596–598. 877576 10.1126/science.877576

[B29] Creese I. Snyder S. H. (1979). Nigrostriatal lesions enhance striatal 3H-apomorphine and 3H-spiroperidol binding. Eur. J. Pharmacol. 56, 277–281. 10.1016/0014-2999(79)90184-5 38973

[B30] Cremer J. N. Amunts K. Graw J. Piel M. Rösch F. Zilles K. (2015). Neurotransmitter receptor density changes in Pitx3ak mice–a model relevant to Parkinson's disease. Neuroscience 285, 11–23. 10.1016/j.neuroscience.2014.10.050 25451278

[B31] Cutson T. Laub K. Schenkman M. (1995). Pharmacological and nonpharmacological interventions in the treatment of Parkinson's disease. Phys. Ther. 75, 363–373. 7732080 10.1093/ptj/75.5.363

[B32] Dautan D. Huerta-Ocampo I. Witten I. B. Deisseroth K. Bolam J. P. Gerdjikov T.. (2014). A major external source of cholinergic innervation of the striatum and nucleus accumbens originates in the brainstem. J. Neurosci. 34, 4509–4518. 10.1523/JNEUROSCI.5071-13.2014 24671996 PMC3965779

[B33] Dawson T. M. Dawson V. L. Gage F. H. Fisher L. J. Hunt M. A. Wamsley J. K. (1991). Downregulation of muscarinic receptors in the rat caudate-putamen after lesioning of the ipsilateral nigrostriatal dopamine pathway with 6-hydroxydopamine (6-OHDA): normalization by fetal mesencephalic transplants. Brain Res. 540, 145–152. 10.1016/0006-8993(91)90501-L 1905173

[B34] DeBoer P. Abercrombie E. D. Heeringa M. Westerink B. H. C. (1993). Differential effect of systemic administration of bromocriptine and l-DOPA on the release of acetylcholine from striatum of intact and 6-OHDA-treated rats. Brain Res. 608, 198–203. 10.1016/0006-8993(93)91459-6 8495354

[B35] De Klippel N. Sarre S. Ebinger G. Michotte Y. (1993). Effect of M1- and M2-muscarinic drugs on striatal dopamine release and metabolism: an *in vivo* microdialysis study comparing normal and 6-hydroxydopamine-lesioned rats. Brain Res. 630, 57–64. 8118706 10.1016/0006-8993(93)90642-z

[B36] Ding J. Guzman J. N. Tkatch T. Chen S. Goldberg J. A. Ebert P. J.. (2006). RGS4-dependent attenuation of M4 autoreceptor function in striatal cholinergic interneurons following dopamine depletion. Nat. Neurosci. 9, 832–842. 10.1038/nn1700 16699510

[B37] Duty S. Jenner P. (2011). Animal models of Parkinson's disease: a source of novel treatments and clues to the cause of the disease. Br. J. Pharmacol. 164, 1357–1391. 10.1111/j.1476-5381.2011.01426.x 21486284 PMC3229766

[B38] Duvoisin R. C. (1967). Cholinergic-anticholinergic antagonism in parkinsonism. Arch. Neurol. 17, 124–136. 4382112 10.1001/archneur.1967.00470260014002

[B39] El-Bizri H. Clarke P. B. S. (1994). Blockade of nicotinic receptor-mediated release of dopamine from striatal synaptosomes by chlorisondamine and other nicotinic antagonists administered *in vitro*. Br. J. Pharmacol. 111, 406–413. 10.1111/j.1476-5381.1994.tb14749.x 8004384 PMC1909987

[B40] Fernandez H. H. (2012). Updates in the medical management of Parkinson disease. Cleve. Clin. J. Med. 79, 28–35. 10.3949/ccjm.78gr.11005 22219231

[B41] Giorguieff M. F. Le Floc'h M. L. Westfall T. C. Glowinski J. Besson M. J. (1976). Nicotinic effect of acetylcholine on the release of newly synthesized (3H)dopamine in rat striatal slices and cat caudate nucleus. Brain Res. 106, 117–131. 1268701 10.1016/0006-8993(76)90077-9

[B42] Giraldo E. Hammer R. Ladinsky H. (1987). Distribution of muscarinic receptor subtypes in rat brain as determined in binding studies with AF-DX 116 and pirenzepine. Life Sci. 40, 833–840. 3821380 10.1016/0024-3205(87)90031-2

[B43] Goldberg J. A. Ding J. B. Surmeier D. J. (2012). Muscarinic modulation of striatal function and circuitry. Handb. Exp. Pharmacol. 208, 223–241. 10.1007/978-3-642-23274-9_10 22222701

[B44] Grady S. Marks M. J. Wonnacott S. Collins A. C. (1992). Characterization of nicotinic receptor-mediated [3H]dopamine release from synaptosomes prepared from mouse striatum. J. Neurochem. 59, 848–856. 1494911 10.1111/j.1471-4159.1992.tb08322.x

[B45] Haghir H. Kovac S. Speckmann E. J. Zilles K. Gorji A. (2009). Patterns of neurotransmitter receptor distributions following cortical spreading depression. Neuroscience 163, 1340–1352. 10.1016/j.neuroscience.2009.07.067 19665048

[B46] Happe H. K. Peters J. L. Bergman D. Murrin L. C. (1994). Localizationof nicotinic cholinergic receptors in rat brain: autoradiographic studies with [3H]cytisine. Neuroscience 62, 929–944. 10.1016/0306-4522(94)90484-7 7870314

[B47] Havekes R. Abel T. Van der Zee E. A. (2011). The cholinergic system and neostriatal memory functions. Behav. Brain Res. 221, 412–423. 10.1016/j.bbr.2010.11.047 21129408 PMC3075367

[B48] Hawlitschka A. Antipova V. Schmitt O. Witt M. Benecke R. Mix E.. (2013). Intracerebrally applied botulinum neurotoxin in experimental neuroscience. Curr. Pharm. Biotechnol. 14, 124–130. 10.2174/138920113804805331 23092264

[B49] Hefco V. Yamada K. Hefco A. Hritcu L. Tiron A. Nabeshima T. (2004). The interaction between the cholinergic and dopaminergic system in learning and memory process in rats. Rom. J. Physiol. 41, 21–30. 15984653

[B50] Hersch S. M. Gutekunst C. A. Rees H. D. Heilman C. J. Levey A. I. (1994). Distribution of m1-m4 muscarinic receptor proteins in the rat striatum: light and electron microscopic immunocytochemistry using subtype-specific antibodies. J. Neurosci. 14, 3351–3363. 10.1523/JNEUROSCI.14-05-03351.1994 8182478 PMC6577507

[B51] Holzmann C. Dräger D. Mix E. Hawlitschka A. Antipova V. Benecke R.. (2012). Effects of intrastriatal botulinum neurotoxin a on the behavior of Wistar rats. Behav. Brain Res. 234, 107–116. 10.1016/j.bbr.2012.06.008 22728288

[B52] Hornykiewicz O. (1963). The tropical localization and content of noradrenalin and dopamine (3-hydroxytyramine) in the substantia nigra of normal persons and patients with Parkinson's disease. Wien. Klin. Wochenschr. 75, 309–312. 13954967

[B53] Horstink M. Tolosa E. Bonuccelli U. Deuschl G. Friedman A. Kanovsky P.. (2006a). Review of the therapeutic management of Parkinson's disease. report of a joint task force of the European Federation of Neurological Societies (EFNS) and the Movement Disorder Society-European Section (MDS-ES). Part II: late (complicated) Parkinson's disease. Eur. J. Neurol. 13, 1186–1202. 10.1111/j.1468-1331.2006.01548.x 17038032

[B54] Horstink M. Tolosa E. Bonuccelli U. Deuschl G. Friedman A. Kanovsky P.. (2006b). Review of the therapeutic management of Parkinson's disease. report of a joint task force of the European Federation of Neurological Societies and the Movement Disorder Society-European Section. Part I: early (uncomplicated) Parkinson's disease. Eur. J. Neurol. 13, 1170–1185. 10.1111/j.1468-1331.2006.01547.x 17038031

[B55] Hunt S. Schmidt J. (1978). Some observations on the binding patterns of alpha-Bungarotoxin in the central nervous system of the rat. Brain Res. 157, 213–232. 10.1016/0006-8993(78)90025-2 719523

[B56] Hyland B. I. Reynolds J. N. J. Hay J. Perk C. G. Miller R. (2002). Firing modes of midbrain dopamine cells in the freely moving rat. Neuroscience 114, 475–492. 10.1016/S0306-4522(02)00267-1 12204216

[B57] Ince E. Ciliax B. J. Levey A. I. (1997). Differential expression of D1 and D2 dopamine and m4 muscarinic acetylcholine receptor proteins in identified striatonigral neurons. Synapse 27, 357–366. 10.1002/(SICI)1098-2396(199712)27:4<357::AID-SYN9>3.0.CO;2-B 9372558

[B58] Jakubík J. Randáková A. Zimčík P. El-Fakahany E. E. DoleŽal V. (2017). Binding of N-methylscopolamine to the extracellular domain of muscarinic acetylcholine receptors. Sci. Rep. 7:40381. 10.1038/srep40381 28091608 PMC5238504

[B59] Jones I. W. Bolam J. P. Wonnacott S. (2001). Presynaptic localisation of the nicotinic acetylcholine receptor beta2 subunit immunoreactivity in rat nigrostriatal dopaminergic neurones. J. Comp. Neurol. 439, 235–247. 10.1002/cne.1345 11596051

[B60] Joyce J. N. (1991). Differential response of striatal dopamine and muscarinic cholinergic receptor subtypes to the loss of dopamine. Exp. Neurol. 113, 261–276. 10.1016/0014-4886(91)90016-6 1833219

[B61] Kaiser S. Wonnacott S. (2000). α-Bungarotoxin-Sensitive nicotinic receptors indirectly modulate [3H]dopamine release in rat striatal slices via glutamate release. Mol. Pharmacol. 58, 312–318. 10.1124/mol.58.2.312 10908298

[B62] Katzenschlager R. Lees A. J. (2002). Treatment of Parkinson's disease: levodopa as the first choice. J. Neurol. 249(Suppl. 2), II19–II24. 10.1007/s00415-002-1204-4 12375059

[B63] Kawaguchi Y. (1993). Physiological, morphological, and histochemical characterization of three classes of interneurons in rat neostriatum. J. Neurosci. 13, 4908–4923. 7693897 10.1523/JNEUROSCI.13-11-04908.1993 PMC6576359

[B64] Kawaguchi Y. Wilson C. J. Augood S. J. Emson P. C. (1995). Striatal interneurones: chemical, physiological and morphological characterization. Trends Neurosci. 18, 527–535. 8638293 10.1016/0166-2236(95)98374-8

[B65] Knol R. J. de Bruin K. Opmeer B. Voorn P. Jonker A. J. van Eck-Smit B. L. F.. (2014). Decreased ipsilateral [^123^I]iododexetimide binding to cortical muscarinic receptors in unilaterally 6-hydroxydopamine lesioned rats. Nucl. Med. Biol. 41, 90–95. 10.1016/j.nucmedbio.2013.10.003 24267055

[B66] Kulak J. M. McIntosh J. M. Quik M. (2002). Loss of nicotinic receptors in monkey striatum after 1-methyl-4-phenyl-1,2,3,6-tetrahydropyridine treatment is due to a decline in alpha-conotoxin MII sites. Mol. Pharmacol. 61, 230–238. 10.1124/mol.61.1.230 11752225

[B67] Lange K. Wells F. Jenner P. Marsden C. (1993). Altered muscarinic and nicotinic receptor densities in cortical and subcortical brain regions in Parkinson's disease. J. Neurochem. 60, 197–203. 8417140 10.1111/j.1471-4159.1993.tb05838.x

[B68] Langmead C. J. Watson J. Reavill C. (2008). Muscarinic acetylcholine receptors as CNS drug targets. Pharmacol. Ther. 117, 232–243. 10.1016/j.pharmthera.2007.09.009 18082893

[B69] Lees A. (2005). Alternatives to levodopa in the initial treatment of early Parkinson's disease. Drugs Aging 22, 731–740. 10.2165/00002512-200522090-00002 16156677

[B70] Lester D. B. Rogers T. D. Blaha C. D. (2010). Acetylcholine-dopamine interactions in the pathophysiology and treatment of CNS disorders. CNS Neurosci. Ther. 16, 137–162. 10.1111/j.1755-5949.2010.00142.x 20370804 PMC6493877

[B71] Levey A. I. Edmunds S. M. Heilman C. J. Desmond T. J. Frey K. A. (1994). Localization of muscarinic M3 receptor protein and M3 receptor binding in rat brain. Neuroscience 63, 207–221. 10.1016/0306-4522(94)90017-5 7898649

[B72] Levey A. I. Kitt C. A. Simonds W. F. Price D. L. Brann M. R. (1991). Identification and localization of muscarinic acetylcholine receptor proteins in brain with subtype-specific antibodies. J. Neurosci. 11, 3218–3226. 1941081 10.1523/JNEUROSCI.11-10-03218.1991 PMC6575445

[B73] Lindefors N. Ungerstedt U. (1990). Bilateral regulation of glutamate tissue and extracellular levels in caudate-putamen by midbrain dopamine neurons. Neurosci. Lett. 115, 248–252. 1978265 10.1016/0304-3940(90)90463-j

[B74] López-Giménez J. F. Mengod G. Palacios J. M. Vilaró M. T. (1997). Selective visualization of rat brain 5-HT(2A) receptors by autoradiography with [3H]MDL 100,907. Naunyn. Schmiedebergs Arch. Pharmacol. 356, 446–454. 10.1007/PL00005075 9349630

[B75] Mailman R. B. (1983). Lithium-induced polydipsia: dependence on nigrostriatal dopamine pathway and relationship to changes in the renin-angiotensin system. Psychopharmacology (Berl) 80, 143–149. 6410443 10.1007/BF00427958

[B76] Mann T. Kurth J. Hawlitschka A. Stenzel J. Lindner T. Polei S. (2018a). [18F]fallypride-PET/CT Analysis of the dopamine D2/D3 receptor in the hemiparkinsonian rat brain following intrastriatal botulinum neurotoxin an injection. Molecules 23:piiE587 10.3390/molecules23030587 PMC6017015 29509680

[B77] Mann T. Zilles K. Dikow H. Hellfritsch A. Cremer M. Piel M.. (2018b). Dopamine, Noradrenaline and Serotonin Receptor Densities in the Striatum of Hemiparkinsonian Rats following Botulinum Neurotoxin-A Injection. Neuroscience 374, 187–204. 10.1016/j.neuroscience.2018.01.053 29421436

[B78] Marti M. Sbrenna S. Fuxe K. Bianchi C. Beani L. Morari M. (1999). *In vitro* evidence for increased facilitation of striatal acetylcholine release via pre- and postsynaptic NMDA receptors in hemiparkinsonian rats. J. Neurochem. 72, 875–878. 9930765 10.1046/j.1471-4159.1999.720875.x

[B79] Mash D. C. Potter L. T. (1986). Localization of ml and m2 muscarine receptors in the rat brain. Neuroscience 19, 551–564. 10.1016/0306-4522(86)90280-0 3774154

[B80] Maurice N. Mercer J. Chan C. S. Hernandez-Lopez S. Held J. Tkatch T.. (2004). D2 dopamine receptor-mediated modulation of voltage-dependent Na+ channels reduces autonomous activity in striatal cholinergic interneurons. J. Neurosci. 24, 10289–10301. 10.1523/JNEUROSCI.2155-04.2004 15548642 PMC6730305

[B81] McOmish C. Pavey G. McLean C. Horne M. Dean B. Scarr E. (2017). Muscarinic receptor binding changes in postmortem Parkinson's disease. J. Neural Transm. 124, 227–236. 10.1007/s00702-016-1629-z 27873015

[B82] Mehlan J. Brosig H. Schmitt O. Mix E. Wree A. Hawlitschka A. (2016). Intrastriatal injection of botulinum neurotoxin-a is not cytotoxic in rat brain - A histological and stereological analysis. Brain Res. 1630, 18–24. 10.1016/j.brainres.2015.10.056 26562665

[B83] Mesulam M.-M. (2013). Cholinergic circuitry of the human nucleus basalis and its fate in Alzheimer's disease. J. Comp. Neurol. 521, 4124–4144. 10.1002/cne.23415 23852922 PMC4175400

[B84] Meyer P. M. Strecker K. Kendziorra K. Becker G. Hesse S. Woelpl D.. (2009). Reduced alpha4beta2^*^-nicotinic acetylcholine receptor binding and its relationship to mild cognitive and depressive symptoms in Parkinson disease. Arch. Gen. Psychiatry 66, 866–877. 10.1001/archgenpsychiatry.2009.106 19652126

[B85] Muccioli G. Raso G. M. Ghé C. Di Carlo R. (1996). Effect of L-alpha glycerylphosphorylcholine on muscarinic receptors and membrane microviscosity of aged rat brain. Prog. Neuropsychopharmacol. Biol. Psychiatry 20, 323–339. 8861196 10.1016/0278-5846(95)00313-4

[B86] Muma N. A. Lee J. M. Gorman L. Heidenreich B. A. Mitrovic I. Napier T. C. (2001). 6-hydroxydopamine-induced lesions of dopaminergic neurons alter the function of postsynaptic cholinergic neurons without changing cytoskeletal proteins. Exp. Neurol. 168, 135–143. 10.1006/exnr.2000.7582 11170728

[B87] Murrin L. C. Gale K. Kuhar M. J. (1979). Autoradiographic localization of neuroleptic and dopamine receptors in the caudate-putamen and substantial nigra: effects of lesions. Eur. J. Pharmacol. 60, 229–235. 10.1016/0014-2999(79)90222-X 43260

[B88] Nash J. E. Brotchie J. M. (2002). Characterisation of striatal NMDA receptors involved in the generation of parkinsonian symptoms: intrastriatal microinjection studies in the 6-OHDA-lesioned rat. Mov. Disord. 17, 455–466. 10.1002/mds.10107 12112191

[B89] Newman E. L. Gupta K. Climer J. R. Monaghan C. K. Hasselmo M. E. (2012). Cholinergic modulation of cognitive processing: insights drawn from computational models. Front. Behav. Neurosci. 6:24. 10.3389/fnbeh.2012.00024 22707936 PMC3374475

[B90] Oorschot D. E. (1996). Total number of neurons in the neostriatal, pallidal, subthalamic, and substantia nigral nuclei of the rat basal ganglia: a stereological study using the cavalieri and optical disector methods. J. Comp. Neurol. 366, 580–599. 10.1002/(SICI)1096-9861(19960318)366:4<580::AID-CNE3>3.0.CO;2-0 8833111

[B91] Pakhotin P. Bracci E. (2007). Cholinergic interneurons control the excitatory input to the striatum. J. Neurosci. 27, 391–400. 10.1523/JNEUROSCI.3709-06.2007 17215400 PMC6672079

[B92] Paxinos G. Watson C. (2007). The Rat Brain in Stereotaxic Coordinates, 6th Edn. San Diego, CA: Academic press.

[B93] Perez X. A. Bordia T. McIntosh J. M. Quik M. (2010). α6ß2^*^ and α4ß2^*^ nicotinic receptors both regulate dopamine signaling with increased nigrostriatal damage: relevance to Parkinson's disease. Mol. Pharmacol. 78, 971–980. 10.1124/mol.110.067561 20732972 PMC2981368

[B94] Pisani A. Bonsi P. Centonze D. Gubellini P. Bernardi G. Calabresi P. (2003). Targeting striatal cholinergic interneurons in Parkinson's disease: Focus on metabotropic glutamate receptors. Neuropharmacology 45, 45–56. 10.1016/S0028-3908(03)00137-0 12814658

[B95] Pradhan A. A. A. Cumming P. Clarke P. B. S. (2002). [125I]Epibatidine-labelled nicotinic receptors in the extended striatum and cerebral cortex: lack of association with serotonergic afferents. Brain Res. 954, 227–236. 10.1016/S0006-8993(02)03340-1 12414106

[B96] Przedbroski S. Leviver M. Jiang H. Ferreira M. Jackson-Lewis V. Donaldson D. (1995). Dose-dependent lesions of the dopaminergic nigrostriatal pathway induced by instrastriatal injection of 6-hydroxydopamine. Neuroscience 67, 631–647. 10.1016/0306-4522(95)00066-R 7675192

[B97] Raiteri M. Leardi R. Marchi M. (1984). Heterogeneity of presynaptic muscarinic receptors regulating neurotransmitter release in the rat brain. J. Pharmacol. Exp. Ther. 228, 209–214. 6141277

[B98] Rakovska A. Javitt D. Raichev P. Ang R. Balla A. Aspromonte J.. (2003). Physiological release of striatal acetylcholine (*in vivo*): effect of somatostatin on dopaminergic-cholinergic interaction. Brain Res. Bull. 61, 529–536. 10.1016/S0361-9230(03)00192-8 13679252

[B99] Rapier C. Lunt G. G. Wonnacott S. (1988). Stereoselective nicotine-induced release of dopamine from striatal synaptosomes: concentration dependence and repetitive stimulation. J. Neurochem. 50, 1123–1130. 3346670 10.1111/j.1471-4159.1988.tb10582.x

[B100] Rice M. E. Cragg S. J. (2004). Nicotine amplifies reward-related dopamine signals in striatum. Nat. Neurosci. 7, 583–584. 10.1038/nn1244 15146188

[B101] Schoffelmeer A. N. Van Vliet B. J. Wardeh G. Mulder A. H. (1986). Muscarine receptor-mediated modulation of [3H]dopamine and [14C]acetylcholine release from rat neostriatal slices: selective antagonism by gallamine but not pirenzepine. Eur. J. Pharmacol. 128, 291–294. 3792444 10.1016/0014-2999(86)90781-8

[B102] Schwarting R. K. Huston J. P. (1996). The unilateral 6-hydroxydopamine lesion model in behavioral brain research. analysis of functional deficits, recovery and treatments. Prog. Neurobiol. 50, 275–331. 8971983 10.1016/s0301-0082(96)00040-8

[B103] Schwartz R. D. Kellar K. J. (1983). Nicotinic cholinergic receptor binding sites in the brain: regulation *in vivo*. Science 220, 214–216. 6828889 10.1126/science.6828889

[B104] Sharma V. K. Harik S. I. Busto R. Banerjee S. P. (1981). Effects of noradrenaline depletion on adrenergic and muscarinic cholinergic receptors in the cerebral cortex, hippocampus, and cerebellum. Exp. Neurol. 72, 179–194. 6258960 10.1016/0014-4886(81)90136-9

[B105] Sharples C. G. Kaiser S. Soliakov L. Marks M. J. Collins A. C. Washburn M.. (2000). UB-165: a novel nicotinic agonist with subtype selectivity implicates the alpha4beta2^*^ subtype in the modulation of dopamine release from rat striatal synaptosomes. J. Neurosci. 20, 2783–2791. 10.1523/JNEUROSCI.20-08-02783.2000 10751429 PMC6772190

[B106] Sivam S. P. Norris J. C. Lim D. K. Hoskins B. Ho I. K. (1983). Effect of acute and chronic cholinesterase inhibition with diisopropylfluorophosphate on muscarinic, dopamine, and GABA receptors of the rat striatum. J. Neurochem. 40, 1414–1422. 6300336 10.1111/j.1471-4159.1983.tb13584.x

[B107] Spehlmann R. Stahl S. M. (1976). Dopamine acetylcholine imbalance in Parkinson's disease. Possible regenerative overgrowth of cholinergic axon terminals. Lancet 1, 724–726. 56538 10.1016/s0140-6736(76)93095-6

[B108] Staunton D. A. Wolfe B. B. Groves P. M. Molinoff P. B. (1981). Dopamine receptor changes following destruction of the nigrostriatal pathway: lack of a relationship to rotational behavior. Brain Res. 211, 315–327. 10.1016/0006-8993(81)90704-6 7237126

[B109] Sugita S. Uchimura N. Jiang Z. G. North R. A. (1991). Distinct muscarinic receptors inhibit release of gamma-aminobutyric acid and excitatory amino acids in mammalian brain. Proc. Natl. Acad. Sci. U.S.A. 88, 2608–2611. 1672454 10.1073/pnas.88.6.2608 PMC51282

[B110] Sun W. Sugiyama K. Asakawa T. Yamaguchi H. Akamine S. Ouchi Y.. (2011). Dynamic changes of striatal dopamine D2 receptor binding at later stages after unilateral lesions of the medial forebrain bundle in Parkinsonian rat models. Neurosci. Lett. 496, 157–162. 10.1016/j.neulet.2011.04.006 21514359

[B111] Svensson A. L. Alafuzoff I. Nordberg A. (1992). Characterization of muscarinic receptor subtypes in Alzheimer and control brain cortices by selective muscarinic antagonists. Brain Res. 596, 142–148. 1467980 10.1016/0006-8993(92)91541-l

[B112] Threlfell S. Clements M. A. Khodai T. Pienaar I. S. Exley R. Wess J.. (2010). Striatal muscarinic receptors promote activity dependence of dopamine transmission via distinct receptor subtypes on cholinergic interneurons in ventral versus dorsal striatum. J. Neurosci. 30, 3398–3408. 10.1523/jneurosci.5620-09.2010 20203199 PMC2866006

[B113] Tieu K. (2011). A guide to neurotoxic animal models of Parkinson's disease. Cold Spring Harb. Perspect. Med. 1:a009316. 10.1101/cshperspect.a009316 22229125 PMC3234449

[B114] Ungerstedt U. (1968). 6-Hydroxy-dopamine induced degeneration of central monoamine neurons. Eur. J. Pharmacol. 5, 107–110. 10.1016/0014-2999(68)90164-7 5718510

[B115] Ungerstedt U. Arbuthnott G. W. (1970). Quantitative recording of rotational behavior in rats after 6-hydroxy-dopamine lesions of the nigrostriatal dopamine system. Brain Res. 24, 485–493. 10.1016/0006-8993(70)90187-3 5494536

[B116] Ungerstedt U. Butcher L. L. Butcher S. G. Andén N. E. Fuxe K. (1969). Direct chemical stimulation of dopaminergic mechanisms in the neostriatum of the rat. Brain Res. 14, 461–471. 4893861 10.1016/0006-8993(69)90122-x

[B117] Valuskova P. Farar V. Forczek S. Krizova I. Myslivecek J. (2018). Autoradiography of 3H-pirenzepine and 3H-AFDX-384 in Mouse Brain Regions: possible insights into M1, M2, and M4 muscarinic receptors distribution. Front. Pharmacol. 9:124. 10.3389/fphar.2018.00124 29515448 PMC5826229

[B118] Volpato C. Schiff S. Facchini S. Silvoni S. Cavinato M. Piccione F.. (2016). Dopaminergic Medication Modulates Learning from Feedback and Error-Related Negativity in Parkinson's Disease: A Pilot Study. Front. Behav. Neurosci. 10:205. 10.3389/fnbeh.2016.00205 27822182 PMC5075574

[B119] Wang Q. Wei X. Gao H. Li J. Liao J. Liu X.. (2014). Simvastatin reverses the downregulation of M1/4 receptor binding in 6-hydroxydopamine-induced parkinsonian rats: the association with improvements in long-term memory. Neuroscience 267, 57–66. 10.1016/j.neuroscience.2014.02.031 24613723

[B120] Wedekind F. Oskamp A. Lang M. Hawlitschka A. Zilles K. Wree A. (2018). Intrastriatal administration of botulinum neurotoxin A normalizes striatal D2 R binding and reduces striatal D1 R binding in male hemiparkinsonian rats. J. Neurosci. Res. 96, 75–86. 10.1002/jnr.24110 28695985

[B121] Westfall T. C. (1974). Effect of muscarinic agonists on the release of 3H-norepinephrine and 3H-dopamine by potassium and electrical stimulation from rat brain slices. Life Sci. 14, 1641–1652. 4407738 10.1016/0024-3205(74)90266-5

[B122] Wonnacott S. Kaiser S. Mogg A. Soliakov L. Jones I. W. (2000). Presynaptic nicotinic receptors modulating dopamine release in the rat striatum. Eur. J. Pharmacol. 393, 51–58. 10.1016/S0014-2999(00)00005-4 10770997

[B123] Woolf N. J. Butcher L. L. (1981). Cholinergic neurons in the caudate-putamen complex proper are intrinsically organized: a combined Evans blue and acetylcholinesterase analysis. Brain Res. Bull. 7, 487–507. 7317794 10.1016/0361-9230(81)90004-6

[B124] Woolf N. J. Butcher L. L. (1986). Cholinergic systems in the rat brain: III. projections from the pontomesencephalic tegmentum to the thalamus, tectum, basal ganglia, and basal forebrain. Brain Res. Bull. 16, 603–637. 3742247 10.1016/0361-9230(86)90134-6

[B125] Wree A. Mix E. Hawlitschka A. Antipova V. Witt M. Schmitt O.. (2011). Intrastriatal botulinum toxin abolishes pathologic rotational behaviour and induces axonal varicosities in the 6-OHDA rat model of Parkinson's disease. Neurobiol. Dis. 41, 291–298. 10.1016/j.nbd.2010.09.017 20955797

[B126] Xu M. Mizobe F. Yamamoto T. Kato T. (1989). Differential effects of M1- and M2-muscarinic drugs on striatal dopamine release and metabolism in freely moving rats. Brain Res. 495, 232–242. 2765928 10.1016/0006-8993(89)90217-5

[B127] Yamada S. Isogai M. Okudaira H. Hayashi E. (1983). Correlation between cholinesterase inhibition and reduction in muscarinic receptors and choline uptake by repeated diisopropylfluorophosphate administration: antagonism by physostigmine and atropine. J. Pharmacol. Exp. Ther. 226, 519–525. 6875862

[B128] Yan Z. Flores-Hernandez J. Surmeier D. J. (2001). Coordinated expression of muscarinic receptor messenger RNAs in striatal medium spiny neurons. Neuroscience 103, 1017–1024. 10.1016/S0306-4522(01)00039-2 11301208

[B129] Yung K. K. L. Bolam J. P. Smith A. D. Hersch S. M. Ciliax B. J. Levey A. I. (1995). Immunocytochemical localization of D1 and D2 dopamine receptors in the basal ganglia of the rat: light and electron microscopy. Neuroscience 65, 709–730. 10.1016/0306-4522(94)00536-E 7609871

[B130] Zhang H. Sulzer D. (2004). Frequency-dependent modulation of dopamine release by nicotine. Nat. Neurosci. 7, 581–582. 10.1038/nn1243 15146187

[B131] Zhang W. Yamada M. Gomeza J. Basile A. S. Wess J. (2002). Multiple muscarinic acetylcholine receptor subtypes modulate striatal dopamine release, as studied with M1-M5 muscarinic receptor knock-out mice. J. Neurosci. 22, 6347–6352. 10.1523/JNEUROSCI.22-15-06347.2002 12151512 PMC6758135

[B132] Zhou F. M. Liang Y. Dani J. A. (2001). Endogenous nicotinic cholinergic activity regulates dopamine release in the striatum. Nat. Neurosci. 4, 1224–1229. 10.1038/nn769 11713470

[B133] Zhou F.-M. Wilson C. Dani J. A. (2003). Muscarinic and nicotinic cholinergic mechanisms in the mesostriatal dopamine systems. Neuroscientist 9, 23–36. 10.1177/1073858402239588 12580337

[B134] Zilles K. Gross G. Schleicher A. Schildgen S. Bauer A. Bahro M.. (1991a). Regional and laminar distributions of alpha 1-adrenoceptors and their subtypes in human and rat hippocampus. Neuroscience 40, 307–320. 1674110 10.1016/0306-4522(91)90122-5

[B135] Zilles K. Palomero-Gallagher N. Grefkes C. Scheperjans F. Boy C. Amunts K.. (2002a). Architectonics of the human cerebral cortex and transmitter receptor fingerprints: Reconciling functional neuroanatomy and neurochemistry. Eur. Neuropsychopharmacol. 12, 587–599. 10.1016/S0924-977X(02)00108-6 12468022

[B136] Zilles K. Qü M. S. Schröder H. Schleicher A. (1991b). Neurotransmitter receptors and cortical architecture. J. Hirnforsch. 32, 343–356. 1663963

[B137] Zilles K. Schleicher A. Palomero-Gallagher N. Amunts K. (2002b). Quantitative analysis of cyto- and receptor architecture of the human brain. Brain Mapp. Methods 58, 573–602. 10.1016/B978-012693019-1/50023-X

[B138] Zilles K. Werner L. Qü M. Schleicher A. Gross G. (1991c). Quantitative autoradiography of 11 different transmitter binding sites in the basal forebrain region of the rat–evidence of heterogeneity in distribution patterns. Neuroscience 42, 473–481. 1654535 10.1016/0306-4522(91)90390-a

[B139] Zoli M. Moretti M. Zanardi A. McIntosh J. M. Clementi F. Gotti C. (2002). Identification of the nicotinic receptor subtypes expressed on dopaminergic terminals in the rat striatum. J. Neurosci. 22, 8785–8789. 10.1523/JNEUROSCI.22-20-08785.2002 12388584 PMC6757689

[B140] Zubieta J. K. Frey K. A. (1993). Autoradiographic mapping of M3 muscarinic receptors in the rat brain. J. Pharmacol. Exp. Ther. 264, 415–422. 8423541

